# Strand annealing and motor driven activities of SMARCAL1 and ZRANB3 are stimulated by RAD51 and the paralog complex

**DOI:** 10.1093/nar/gkac583

**Published:** 2022-07-08

**Authors:** Swagata Halder, Lepakshi Ranjha, Angelo Taglialatela, Alberto Ciccia, Petr Cejka

**Affiliations:** Institute for Research in Biomedicine, Università della Svizzera italiana (USI), Faculty of Biomedical Sciences, Bellinzona, Switzerland; Institute for Research in Biomedicine, Università della Svizzera italiana (USI), Faculty of Biomedical Sciences, Bellinzona, Switzerland; Department of Genetics and Development, Herbert Irving Comprehensive Cancer Center, Columbia University Irving Medical Center, NY, USA; Department of Genetics and Development, Herbert Irving Comprehensive Cancer Center, Columbia University Irving Medical Center, NY, USA; Institute for Research in Biomedicine, Università della Svizzera italiana (USI), Faculty of Biomedical Sciences, Bellinzona, Switzerland; Department of Biology, Institute of Biochemistry, Eidgenössische Technische Hochschule (ETH), Zürich, Switzerland

## Abstract

SMARCAL1, ZRANB3 and HLTF are required for the remodeling of replication forks upon stress to promote genome stability. RAD51, along with the RAD51 paralog complex, were also found to have recombination-independent functions in fork reversal, yet the underlying mechanisms remained unclear. Using reconstituted reactions, we build upon previous data to show that SMARCAL1, ZRANB3 and HLTF have unequal biochemical capacities, explaining why they have non-redundant functions. SMARCAL1 uniquely anneals RPA-coated ssDNA, which depends on its direct interaction with RPA, but not on ATP. SMARCAL1, along with ZRANB3, but not HLTF efficiently employ ATPase driven translocase activity to rezip RPA-covered bubbled DNA, which was proposed to mimic elements of fork reversal. In contrast, ZRANB3 and HLTF but not SMARCAL1 are efficient in branch migration that occurs downstream in fork remodeling. We also show that low concentrations of RAD51 and the RAD51 paralog complex, RAD51B–RAD51C–RAD51D–XRCC2 (BCDX2), directly stimulate the motor-driven activities of SMARCAL1 and ZRANB3 but not HLTF, and the interplay is underpinned by physical interactions. Our data provide a possible mechanism explaining previous cellular experiments implicating RAD51 and BCDX2 in fork reversal.

## INTRODUCTION

RAD51 is a key protein responsible for genome integrity in human cells. RAD51 has well-defined functions in the repair of DNA double-strand breaks (DSBs) by homologous recombination. The DSBs are first resected, generating ssDNA overhangs coated with the single-stranded DNA binding protein replication protein A (RPA). The breast cancer suppressor BRCA2 and the RAD51 paralog complexes, either RAD51B–RAD51C–RAD51D–XRCC2 (BCDX2) or RAD51C–XRCC3 (CX3) then help load RAD51 on the resected DNA to form a filament, displacing RPA. The RAD51 nucleoprotein filament has the capacity to identify, pair and invade homologous DNA, which is used as a repair template in recombination. The strand exchange activity of RAD51 is thus essential for its canonical function in homologous recombination (1).

More recently, it has been discovered that RAD51 has additional, strand exchange independent functions in the metabolism of replication forks upon stress (2,3). When replicating damaged templates, repetitive DNA sequences or DNA at telomeres, during replication-transcription conflicts or upon the overexpression of oncogenes, the forks can uncouple and the leading strand polymerase may transiently stall (4). Upon prolonged stalling, depending on the cellular context, replication can restart by PRIMPOL-mediated repriming, or the forks can undergo reversal (5–7). Fork reversal involves annealing of the two nascent DNA strands yielding a 4-way junction, followed by branch migration (8–10). For a long time, fork reversal was thought to be only a pathological process (11). More recent data however uncovered that depending on the context and cellular genetic background, fork reversal may be beneficial (8,9,12). Fork reversal may in fact limit the extent of ssDNA at stalled forks, provide cells time to deal with the respective challenge, and in this way to prevent DNA breakage or even enable specific post-replicative DNA repair. Indeed, several motor proteins with unique capacities to reverse replication forks have been identified, including but not limited to SMARCAL1, ZRANB3 and HLTF (9,13). Depletion of either of these DNA translocases leads to defects in fork reversal, showing that these enzymes act in a non-redundant manner. Depletion of SMARCAL1 or ZRANB3 results in sensitivity to conditions inducing replication stress and enhancing genome instability, indicating that fork reversal catalyzed by these enzymes in wild type cells can be a protective event (14–19).

In contrast to homologous recombination, the functions of RAD51 under replication stress remain mostly undefined from a mechanistic standpoint. Cellular and electron microscopy experiments suggested that RAD51 promotes fork reversal (10). While the motor proteins SMARCAL1, ZRANB3 or HLTF catalyze fork remodeling by their strand annealing and branch migration activities, how RAD51 facilitates fork reversal remains puzzling. RAD51 function in fork metabolism appears to be distinct from its function in canonical recombination as it is genetically separable (20), and it is independent of BRCA2 (2,21). More recently, the BCDX2 RAD51 paralog complex was also found to promote fork reversal alongside RAD51 in cellular assays (22), yet the underlying mechanism remains similarly unclear.

Once replication fork reverse, they may become vulnerable to pathological degradation. Proteins such as RAD51, BRCA1 and BRCA2 along with a growing list of additional factors are necessary to protect DNA against nucleases such as MRE11, EXO1, DNA2 or MUS81, depending on the genetic background (9). Depletion of BRCA1 or BRCA2 leads to nascent DNA degradation that extends for kilobases in length (2,3). Such DNA degradation is dependent on the proteins implicated in fork reversal, including SMARCAL1, ZRANB3 and HLTF, which suggested that reversed, but not stalled replication forks are primarily subjected to degradation (2,21,23–25). However, nascent DNA degradation is not observed upon depletion of RAD51 itself (10,21). This apparent paradox was explained by a model where RAD51 also promotes fork reversal (10,21). Accordingly, in the absence of RAD51, the substrates for DNA degradation, i.e. reversed forks, are not formed. In the absence of the relevant substrate, the function of RAD51 in fork protection becomes irrelevant, explaining why nascent DNA is stable upon depletion of RAD51 (10,26). The involvement of RAD51 in fork reversal was confirmed by physical analysis of DNA intermediates by electron microscopy (10). Further experiments revealed that the concentrations of RAD51 required for fork reversal and protection are different: while low RAD51 concentrations are sufficient to promote reversal, elevated RAD51 concentrations are necessary for DNA protection (26,27). Understanding the involvement of RAD51 in fork reversal and DNA protection is highly relevant for cancer therapy, as nascent DNA degradation was linked to lethality of BRCA-deficient cells, and restoration of DNA protection is one of the key mechanisms of chemoresistance of BRCA-deficient tumors (28,29).

Here, we use reconstitution biochemistry to study the fork remodeling enzymes SMARCAL1, ZRANB3 and HLTF, and their regulation by RAD51 and the RAD51 paralog complex. Using comparative biochemical analyses, we show that the motor proteins have distinct biochemical activities, and uncover a strand annealing activity of SMARCAL1, which depends on its specific interaction with RPA. We further show that RAD51 and the BCDX2 RAD51 paralog complex directly promote the motor-driven strand annealing of SMARCAL1 and ZRANB3, underpinned by their physical interactions. Together, our data provide insights into the mechanisms underlying the non-catalytic function of RAD51 and RAD51 paralogs in the metabolism of challenged replication forks.

## MATERIALS AND METHODS

### Cloning, expression and purification of recombinant proteins

#### Human SMARCAL1, ZRANB3

Recombinant FLAG-SMARCAL1, FLAG-ZRANB3 and their variants were expressed in insect *Spodoptera frugiperda* 9 (*Sf*9) cells and purified by affinity chromatography, using pFastBac-FLAG-SMARCAL1 and pFastBac-FLAG-ZRANB3 expression constructs (15). Point mutagenesis of the corresponding DNA sequences was carried out by QuikChange II site-directed mutagenesis kit (Agilent Technologies), and the proteins were expressed and purified similarly as the wild type counterparts. Primers used for cloning and mutagenesis are listed in Supplementary Table S1.

#### Human HLTF

The *HLTF* sequence was codon-optimized for *Sf*9 insect cells and synthesized by Synbio Technologies, and cloned using NheI and XmaI sites (New England Biolabs) into pFB-2xMBP-CtIP-10xHis (30) to create pFB-2xMBP-HLTFco-10xHis, replacing the CtIP sequence with that of HLTF. The bacmids and baculoviruses were prepared according to manufacturer's instructions (Bac-to-Bac system, Life Technologies). *Sf*9 cells were transfected using a Trans-IT insect reagent (Mirus Bio). For protein production, *Sf*9 insect cells were seeded at 0.5 × 10^6^ cells per ml 16 h before infection. The cells were then infected with respective baculoviruses and incubated for 52 h at 27°C with constant agitation. Cells were harvested (500 g, 10 min) and washed once with ice cold phosphate buffer saline (PBS). The cell pellets were snap frozen in liquid nitrogen and stored at -80°C. All the subsequent steps were carried out on ice or at 4°C. The pellets were resuspended and incubated for 20 min with continuous stirring in 3 volumes of lysis buffer (50 mM Tris–HCl pH 7.5, 1 mM dithiothreitol [DTT], 5 mM beta-mercaptoethanol [β-ME], 1 mM phenylmethylsulfonyl fluoride [PMSF], 1:400 [v/v] protease inhibitor cocktail [Sigma, P8340], 30 μg/ml leupeptin [Merck]). Next, 50% glycerol was added to reach a final concentration of ∼16% to the cell extract, followed by 6.5% volume of 5 M NaCl (final concentration 305 mM), and further incubated for 30 min with continuous stirring. The cell suspension was centrifuged for 30 min at 48 000 g to obtain soluble extract. The supernatant was transferred to tubes containing pre-equilibrated amylose resin (New England Biolabs, 4 ml/l of *Sf*9 culture) and incubated for 1 h with continuous rotation. The resin was collected by spinning at 2000 g for 2 min and washed extensively batchwise and also on a disposable 10 ml column (ThermoFisher) with amylose wash buffer (50 mM Tris–HCl pH 7.5, 5 mM β-ME, 1 mM PMSF, 10% glycerol, 1 M NaCl). The final wash was performed at 300 mM NaCl. Protein was eluted with amylose elution buffer (50 mM Tris–HCl pH 7.5, 5 mM β-ME, 1 mM PMSF, 10% glycerol, 300 mM NaCl, 10 mM maltose [Sigma]) and the total protein concentration was estimated by Bradford assay. To cleave off the maltose-binding protein (MBP) tag, 1/6 (w/w) of PreScission Protease, with respect to total protein concentration in the eluate, was added and incubated for 1 h at 4°C. The sample was then supplemented with 10 mM imidazole and further passed through pre-equilibrated (amylose elution buffer supplemented with 10 mM imidazole) Ni-NTA agarose resin (Qiagen) on a disposable column for 1 h in flow. The Ni-NTA resin was washed 4-times with Ni-NTA wash buffer (50 mM Tris–HCl pH 7.5, 5 mM β-ME, 1 M NaCl, 10% glycerol, 1 mM PMSF, 40 mM imidazole). Prior to elution, the protein was washed once with the same Ni-NTA wash buffer as above but with 150 mM NaCl. Protein was eluted in the same buffer supplemented with 300 mM imidazole, and subsequently dialyzed (50 mM Tris–HCl pH 7.5, 5 mM β-ME, 100 mM NaCl, 10% glycerol, 0.5 mM PMSF), sub-aliquoted, snap frozen and stored at –80°C for later use.

#### Human RAD51 paralogs complex BCDX2

Sequences for human RAD51 paralogs (*RAD51B, RAD51C, RAD51D, XRCC2*) were codon-optimized for expression in *Sf*9 cells and synthetized by Synbio Technologies. FLAG-RAD51B and 10xHis-RAD51C were cloned into a pFB dual expression vector (ThermoFisher). The multiple cloning site 1 was utilized for FLAG-RAD51B using BamHI and NotI cloning sites and the multiple cloning site 2 was used for 10xHis-RAD51C employing the XmaI and NheI restriction sites, to create pFB-FLAG–RAD51Bco–10xHis–RAD51Cco. RAD51D and XRCC2 were cloned without any affinity tag into the same restriction sites, respectively, to obtain pFB–RAD51Dco–XRCC2co. Baculoviruses expressing RAD51B–RAD51C and RAD51D–XRCC2 were prepared separately and *Sf*9 cells were co-infected with optimized ratios for these viruses to express the BCDX2 complex as a heterotetramer. Cells were harvested 52 h post infection, washed once with cold PBS, and the pellets were frozen in liquid nitrogen and stored at –80 °C until further use. The subsequent steps were carried out on ice or at 4°C. The cell pellet was resuspended in lysis buffer (50 mM Tris–HCl pH 7.5, 2 mM β-ME, 1:400 [v/v] protease inhibitor cocktail [Sigma], 1 mM PMSF, 30 μg/ml leupeptin [Merck], 20 mM imidazole) for 20 min. Then, 50% glycerol was added to a final concentration of ∼16%, followed by 5 M NaCl to a final concentration of 305 mM. The suspension was incubated for additional 30 min with gentle agitation. The total cell extract was centrifuged at 48 000 g for 30 min to obtain soluble extract. The extract was then bound to Ni-NTA resin (Qiagen) for 1 h batchwise followed by extensive washing with Ni-NTA wash buffer (50 mM Tris–HCl pH 7.5, 2 mM β-ME, 300 mM NaCl, 10% glycerol, 1 mM PMSF, 10 μg/ml leupeptin, 20 mM imidazole) both batchwise and on a disposable column. The protein complexes were eluted by Ni-NTA elution buffer (Ni-NTA wash buffer containing 300 mM imidazole). The eluates were diluted 1:6 with a dilution buffer (Ni-NTA elution buffer without imidazole and 0.5 mM β-ME) and bound to FLAG resin (Sigma) pre-equilibrated with dilution buffer in flow with a total contact time of ∼90 min. Protein bound FLAG-resin was washed 3-times with FLAG wash buffer (50 mM Tris–HCl pH 7.5, 0.5 mM β-ME, 150 mM NaCl, 10% glycerol, 1 mM PMSF) and 2 times with the same buffer with 100 mM NaCl before being eluted with a FLAG elution buffer (FLAG wash buffer with 100 mM NaCl and 150 ng/μl 3xFLAG peptide [Sigma]). Complexes were sub-aliquoted, snap frozen and stored at –80°C for later use.

#### *Drosophila* topoisomerase I

To prepare N-terminally truncated *Drosophila* topoisomerase I (catalytic subunit) with 6xHis tag on its C-terminus, the ND423 plasmid (a kind gift from James T. Kadonaga, University of California, San Diego, USA), was transformed in BL21 (DE3) pLysS cells and protein was purified by nickel affinity chromatography(31). The cell pellet from 1 liter culture was resuspended and sonicated in lysis buffer (50 mM Tris–HCl pH 7.5, 1 mM PMSF, 500 mM NaCl, 10% glycerol, 2 mM β-ME, 10 μg/ml leupeptin, 20 mM imidazole) and supplemented with 1:400 protease inhibitor cocktail (Sigma). Soluble extract was obtained by centrifugation at 48,000 g for 30 min and was incubated with pre-equilibrated Ni-NTA resin (Qiagen) for 2 h at 4°C. Next, resin was washed 4 times with Ni-NTA wash buffer (50 mM Tris–HCl pH 7.5, 1 mM PMSF, 500 mM NaCl, 10% glycerol, 2 mM β-ME, 20 mM imidazole). Before elution the resin was washed once with the same Ni-NTA wash buffer as above but with 100 mM NaCl. Protein was eluted with elution buffer (50 mM Tris–HCl pH 7.5, 1 mM PMSF, 100 mM NaCl, 10% glycerol, 0.5 mM β-ME, 300 mM imidazole, 10 μg/ml leupeptin). Peak fractions were pooled and diluted 1:5 in dilution buffer (50 mM Tris–HCl pH 7.5, 0.5 mM β-ME, 100 mM NaCl, 10% glycerol, 1 mM PMSF, 10 μg/ml leupeptin) and the sample was loaded onto pre-equilibrated HiTrap S and HiTrap Heparin columns connected in tandem (GE Healthcare), and washed with 20 ml of dilution buffer. The same buffer with a salt gradient up to 1 M NaCl was used to elute the protein from the HiTrap Heparin column after the HiTrap S column was disconnected. Peak fractions were pooled and dialyzed in dilution buffer for 2 h. Protein was aliquoted, snap-frozen and stored at –80°C.

#### Human RAD51

The *RAD51* sequence was cloned from pTXB3-RAD51 construct (32) into pMALT-P vector BamHI and PstI restriction sites, yielding N-terminal MBP tag, PreScission protease site and RAD51. The RAD51 was expressed in BL21 (DE3) pLysS cells and was supplemented with 0.2% glucose, induced with 0.5 mM IPTG and grown overnight at 18°C. The cells were then pelleted at 2500 g for 15 min at 4°C, washed once with STE buffer (10 mM Tris–HCl pH 8, 500 mM NaCl, 1 mM EDTA), snap-frozen and kept in –80°C until use. The pellets were then resuspended in lysis buffer (50 mM Tris–HCl pH 7.5, 1 mM PMSF, 1 mM DTT, 10% glycerol, 500 mM NaCl, 1:500 protease inhibitor cocktail [Sigma]), sonicated and lysate was clarified by centrifugation at 48 000 g for 30 min. Next, the lysate was incubated with amylose resin for 1 h batchwise at 4°C, washed first with wash buffer I (50 mM Tris–HCl pH 7.5, 1 mM DTT, 10% glycerol, 1 M NaCl) and then with wash buffer II (50 mM Tris–HCl pH 7.5, 1 mM DTT, 10% glycerol, 300 mM NaCl) followed by elution with wash buffer II containing 10 mM maltose. To cleave off the MBP tag, PreScission Protease was added to the eluate and incubated overnight at 4°C (1:5, w/w). Cleaved RAD51 eluate was diluted with 50 mM Tris–HCl pH 7.5 to lower the NaCl concentration to 150 mM. The eluate was then applied to a Hitrap Q column (GE Healthcare). The column was washed sequentially with wash buffer III (20 mM Tris–HCl pH 7.5, 1 mM EDTA, 0.5 mM DTT, 10% glycerol, 150 mM NaCl) and eluted with wash buffer III with 300 mM NaCl. The fractions containing RAD51 were pooled and dialyzed in 20 mM Tris–HCl pH 7.5, 1 mM DTT, 20% glycerol and 100 mM NaCl overnight. The dialyzed protein was aliquoted, snap-frozen and stored at –80°C. Wild type RAD51 was prepared from 4 l of culture and all other variants were prepared from 1 l cultures following the same purification procedure.

#### Human RPA

Recombinant human RPA was expressed from p11d–tRPA construct (a kind gift from M. Wold, University of Iowa) in BL21 (DE3) pLysS cells. Bacterial culture was grown at 37°C (200 RPM) until O.D._600_ = 0.6, induced with 0.4 mM IPTG, and shaken at 18°C (200 RPM) overnight. Bacterial pellet was obtained by centrifugation, washed once with SD Buffer (10 mM Tris pH 8.0, 150 mM NaCl, 1 mM EDTA), snap-frozen and stored at –80°C. Cell lysis, followed by purification using ÄKTA pure (GE Healthcare) using HiTrap Blue HP, HiTrap desalting and HiTrap Q chromatography columns (all GE Healthcare) (33).

#### Human mitochondrial single-stranded DNA binding protein (SSB)

Recombinant mitochondrial SSB was expressed and purified from *E. coli* BL21 cells (34).

### Mass photometry

Mass photometry measurements were performed on a 2MP-0132 mass photometer (Refeyn Ltd). For the measurements, coverslips (No. 1.5 H thickness, 24 × 50 mm, VWR) were cleaned by dipping it into iso-propanol and Milli-Q water followed by drying under a stream of gaseous nitrogen. Subsequently, silicone gaskets (CultureWellTM Reusable Gasket, Grace Bio-Labs) were placed on the cleaned coverslips to create wells for samples. For mass measurements, gaskets were filled with 18 μl protein elution buffer to allow focusing the microscope onto the coverslip surface. Subsequently, 2 μl (50 nM) protein solution was added into the 18 μl droplets and mixed. A movie was recorded for 1 min using the software AcquireMP (Refeyn Ltd). Data analysis was performed using DiscoverMP (Refeyn Ltd). To convert the measured optical reflection-interference contrast into a molecular mass, a known protein size marker (NativeMarkTM Unstained Protein Standard, Invitrogen) was used.

### Preparation of oligonucleotide-based DNA substrates

Oligonucleotides were either 5′-end-labeled with [γ-^32^P]-ATP (Perkin Elmer) and T4 polynucleotide kinase (New England Biolabs), or 3′-end-labeled with [α-^32^P]-dCTP (Perkin Elmer) and terminal transferase (New England Biolabs) enzymes, respectively. The labeled DNA was then purified on a Micro Bio-Spin P-30 Tris chromatography columns (Bio-Rad) (35). Sequences for all oligonucleotides used to obtain the DNA substrates are listed in Supplementary Table S1.

Branch migration substrate was prepared as described previously (36). Briefly, 5′- or 3′-end-labeled 2 μM XO1 was mixed with 2.4 μM XO2 (1:1.2 ratio) in annealing buffer (10 mM Tris–HCl pH 8, 50 mM NaCl, 10 mM MgCl_2_). In parallel, 2 μM each (1:1 ratio) of XO1c.MM2 and XO2c.MM oligonucleotides were similarly combined. The respective mixes were heated for 3 min at 95°C and slowly cooled down to room temperature overnight. The two respective samples were then combined and annealed together (37°C for 30 min), followed by gradual cooling down to room temperature (2 h). Substrate was then stored at –20°C until further use.

Fork reversal substrates were prepared as described earlier (37). Briefly, to create a fork with a leading strand gap, 3′- or 5′-labeled nascent #DC-6 (100 nM final) was annealed with unlabeled parental #DC-2 (120 nM final) in annealing buffer (as above) by heating (3 min at 95 °C) and gradually cooled down to room temperature overnight. Similarly, the complementary half comprising of unlabeled parental #DC-1 (180 nM final) and unlabeled nascent #DC-4 (180 nM final) were separately annealed. These two corresponding halves (#DC-6 + #DC-2 and #DC-1 + #DC-4) were then combined and annealed at 37 °C for 45 min and then cooled down to room temperature during 2 h, and stored at –20°C until further use. To create a fork with a lagging strand gap, 3′- or 5′-labeled nascent #DC-3 (100 nM final) was annealed with unlabeled parental #DC-1 (120 nM final) and the corresponding half containing unlabeled parental #DC-2 (150 nM final) was annealed with unlabeled nascent #DC-5 (150 nM final) oligos. These two halves (#DC-3 + #DC-1 and #DC-2 + #DC-5) were then combined and annealed as above.

### Topoisomerase-coupled annealing assays

The bubbled DNA annealing assay was performed as described (38) with the following modifications. pBluescript II KS (+) plasmid (a kind gift from Marcus Thelen, IRB, Bellinzona, Switzerland) was used as a substrate. 100 ng supercoiled DNA was mixed with 1 μg RPA in TE pH 8.0 in 10 μl volume, and incubated for 45 min at 37°C. Next, 16.5 nM (final) of catalytic domain of *Drosophila* topoisomerase I was added to the reaction mixture and incubated for additional 10 min at 37 °C. Next, annealing buffer (20 mM Tris–HCl pH 7.5, 5 mM MgCl_2_, 1 mM DTT, 0.2 mg/ml BSA, 100 mM NaCl), 2.5 mM ATP (final) and corresponding amounts of proteins (as indicated in figures) or protein storage buffer were added. The final volume was adjusted to 20 μl with water and the reactions were incubated for 30 min at 37 °C. The reactions were terminated by adding 2.5 μl of 5 M NaCl at room temperature for 2 min, followed by 6.5 μl of 2% stop buffer (100 mM Tris–HCl pH 7.5, 150 mM EDTA, 2% SDS [w/v], 30% glycerol, 0.1% bromophenol blue) and 1 μl Proteinase K (14–22 mg/ml, Roche), and incubated 10 min at 37°C. The mixture was resolved by 1% agarose gel electrophoresis in 1× TAE buffer, and DNA was visualized by post-staining with GelRed (Biotium) according to manufacturer's instructions. The gels were then imaged (InGenius3, GeneSys) and quantitated as the fraction of near or fully relaxed DNA using Image J. Graphs were generated by GraphPad Prism software.

### Fork reversal and branch migration assays

The assays were carried out in a reaction buffer containing 20 mM Tris–HCl pH 7.5, 5 mM MgCl_2_, 1 mM DTT, 0.1 mg/ml BSA, 10% glycerol, 2.5 mM ATP (unless indicated otherwise), and 1 nM (fork reversal) or 0.5 nM (branch migration) DNA substrate, with 150 mM NaCl (unless indicated otherwise). Master-mixes were prepared on ice and where indicated, RPA (3 nM) was added to the master-mix for 15 min on ice. 13 μl reaction mixture was then dispersed to individual tubes and supplemented with other recombinant proteins (as indicated) and final volume was adjusted to 15 μl with protein storage buffer. The reactions were continued for additional 30 min at 37°C and terminated by the addition of 5 μl stop buffer (100 mM Tris–HCl pH 7.5, 150 mM EDTA, 0.2% SDS [w/v], 30% glycerol, 0.1% bromophenol blue) and 1 μl Proteinase K (14–22 mg/ml, Roche) and incubated for 10 min at 37°C. Samples were loaded onto 8% polyacrylamide (19:1 acrylamide:bisacrylamide) gels in 1× Tris–borate–EDTA (TBE) (BIO-RAD Mini-PROTEAN system, 1 mm thick) and separated for 60 min at 80 V at room temperature. The gels were dried using a BIO-RAD gel drier on 17 CHR paper (Whatman), and were exposed to storage phosphor screens and scanned using Typhoon FLA 9500 (GE Healthcare) phosphor imager. The gels were quantitated with ImageJ. Graphs were generated by GraphPad Prism software.

### Single-stranded DNA annealing assay

DNA annealing reactions were carried out at 37°C for the times indicated using complementary oligonucleotides X12-3 and X12-4C (please see Supplementary Table S1 for sequences), 1 nM each. The X12-3 oligonucleotide was labeled at the 3′-end. Control reactions were supplemented with protein storage buffer. Reaction master mixes were prepared separately with the respective ssDNA in a buffer containing 25 mM Tris–acetate pH 7.5, 5 mM magnesium acetate, 1 mM DTT, 1 mM ATP and supplemented with 4 nM RPA or SSB where indicated. The two respective master mixes were then incubated for 5 min at 37°C to allow RPA or SSB binding. The two respective mixes containing complementary ssDNA were then combined. Motor proteins and co-factors (when indicated) were added immediately and reaction volume was adjusted with water. The reactions were then incubated at 37°C; 15 μl reaction mixture was withdrawn at the indicated time points into tubes containing 5 μl stop buffer (100 mM Tris–HCl pH 7.5, 150 mM EDTA, 0.2% SDS [w/v], 30% glycerol, 0.1% bromophenol blue) and 1 μl Proteinase K (14–22 mg/ml, Roche). Tubes were kept on ice until the collection of the last time point, and finally transferred to 37°C for 10 min to achieve deproteination. Samples were then loaded onto 8% polyacrylamide (19:1 acrylamide:bisacrylamide) gels in 1× TBE (BIO-RAD Mini-PROTEAN system, 1 mm thick), and processed as described above.

### ATPase assays

ATPase assays were performed in a buffer containing 20 mM Tris–HCl pH 7.5, 5 mM MgCl_2_, 1 mM DTT, 0.1 mg/ml BSA, 10% glycerol, 100 mM NaCl (unless otherwise indicated in the figures), 100 nM ATP, 1 nM of [γ-^32^P] ATP (Perkin Elmer). 2961 bp long supercoiled pBluescript II KS (+) (7 nM, in molecules) or 5 nM unlabeled fork (#DC-1 + #DC-2) or Holliday junction structures ([XO1 + XO2]+ [XO1c.MM2 + XO2c.MM]) were used as a substrate. Recombinant proteins were added on ice and the samples were incubated at 37°C for 60 min. Reactions were stopped with 2 μl of 0.5 M EDTA and separated using TLC plates (Merk) and 0.3 M LiCl and 0.3 M formic acid as a mobile phase. Dried plates were exposed to storage phosphor screens (GE Healthcare) and scanned by a Typhoon FLA 9500 phosphorimager (GE Healthcare). Signals were quantified using ImageJ software. Spontaneous ATP hydrolysis signal (Pi) from no protein lanes were removed as a background and the fraction of ATP hydrolysis was obtained as a normalized value. Graphs were generated by GraphPad Prism software.

### Electrophoretic mobility shift assays

The electrophoretic mobility shift assay (EMSA) to characterize the binding of BCDX2 complex to 70-mer ssDNA (PC210) or dsDNA (PC210 annealed with PC211), 1 nM final; was carried out in 15 μl volume in a binding buffer containing 20 mM Tris–acetate pH 7.5, 1 mM DTT, 1 mM magnesium acetate, 0.1 mg/mL BSA (New England Biolabs) and 1 mM ATP. PC210 was labeled at 5′ end. Reactions were assembled on ice and supplemented with increasing concentrations of BCDX2, and incubated for 30 min at 37°C. The reactions were mixed with 5 μl loading buffer (50% glycerol, 0.1% bromophenol blue) and loaded on 6% polyacrylamide (19:1 acrylamide:bisacrylamide) gels in 1× TBE (BIO-RAD Mini-PROTEAN system, 1 mm thick), and separated for 150 min at 80 V at 4°C. The gels were dried using a BIO-RAD gel drier on 17 CHR paper (Whatman), exposed to storage phosphor screens and scanned using Typhoon FLA 9500 (GE Healthcare) phosphor imager.

### Protein-protein interaction assays

To study the interaction between SMARCAL1 wild type and corresponding SMARCAL1 F→A variants with RAD51 or between ZRANB3 WT, ZRANB3 F→A variants with RAD51, bacterial soluble extract containing MBP-RAD51 was incubated with amylose resin (50 μl, New England Biolabs). The resin was washed with wash buffer I (25 mM Tris–HCl pH 7.5, 0.5 mM DTT, 3 mM EDTA, 100 mM NaCl, 0.2 μg/μl BSA) and incubated with recombinant purified FLAG-SMARCAL1, FLAG-ZRANB3 or the FLAG-BCDX2 complex (all 1 μg) in 150 μl IP buffer (25 mM Tris–HCl pH 7.5, 0.5 mM DTT, 3 mM EDTA, 100 mM NaCl, 0.2 μg/μl BSA, 10% Glycerol) for 1 h at 4°C. The resin with bound proteins was washed 5 times with 1 ml wash buffer III (25 mM Tris–HCl pH 7.5, 1 mM DTT, 3 mM EDTA, 100 mM NaCl, 0.05% Triton X-100), and eluted with wash buffer III (70 μl) containing 30 mM maltose and Avidin (0.11 μg/μl, Sigma) as a stabilizer. Samples were analyzed by Western blotting using anti-MBP primary antibody (MBL, M091-3, 1:1000) against MBP-RAD51 and anti-FLAG primary antibody (Sigma, F3165, 1:1000) against SMARCAL1, ZRANB3 or against RAD51B of the BCDX2 complex, respectively, by standard procedures.

To study interaction between RAD51 and ZRANB3 variants, FLAG-tagged ZRANB3 variants were expressed in *Sf*9 cells, cells were lysed and soluble extract containing the FLAG-ZRANB3 proteins was bound to M2 anti-FLAG affinity resin (50 μl, Sigma). The resin was washed 3-times with 1 ml wash buffer I (25 mM Tris–HCl pH 7.5, 0.5 mM DTT, 3 mM EDTA, 100 mM NaCl, 0.2 μg/μl BSA) and incubated for 1 h at 4°C with recombinant purified RAD51 (1 μg) in 150 μl IP buffer (25 mM Tris–HCl pH 7.5, 0.5 mM DTT, 3 mM EDTA, 100 mM NaCl, 0.2 μg/μl BSA). The resin with bound proteins was washed 5-times with 1 ml wash buffer II (25 mM Tris–HCl pH 7.5, 0.5 mM DTT, 3 mM EDTA, 100 mM NaCl, 0.1% NP40, 0.2 μg/μl BSA), and proteins were eluted with wash buffer II (70 μl) containing 150 ng/μl 3xFLAG peptide (GLPBIO) and Avidin (0.11 μg/μl, Sigma) as a stabilizer. Samples were analyzed by Western blotting using anti-RAD51 primary antibody (Abcam-133534, 1:1000) or by Ponceau staining to show ZRANB3, using standard laboratory procedures.

To study the interaction between ZRANB3 and the BCDX2 complex, or between SMARCAL1 variants and the BCDX2 complex, 1 μg (1 μl) anti-His primary antibody (MBL-D2913) was mixed with 15 μl Dynabeads Protein G (Invitrogen) slurry in a solution containing 150 μl 1X PBS containing 0.05% Tween 20 (PBS-T). The mixture was incubated for 45 min at room temperature with gentle mixing, washed 3 times with 150 μl PBS-T and was further resuspended in 60 μl IP buffer (25 mM Tris–HCl pH 7.5, 0.5 mM DTT, 3 mM EDTA, 100 mM NaCl, 0.2 μg/μl BSA), which was then supplemented with 1 μg recombinant purified BCDX2 complex and incubated for 1 h at 4°C with gentle mixing. The beads were washed 3-times with 150 μl wash buffer (50 mM Tris–HCl pH 7.5, 1 mM DTT, 3 mM EDTA, 100 mM NaCl, 0.05% Triton X-100) and again resuspended in IP buffer. Purified recombinant SMARCAL1 or ZRANB3 (1 μg) was added and incubated for 1 h at 4°C with gentle mixing, and washed 4-times with wash buffer (50 mM Tris–HCl pH 7.5, 100 mM NaCl, 0.05% Triton X-100). Proteins were eluted by heating the beads for 3 min at 95°C in 60 μl SDS buffer (50 mM Tris–HCl pH 6.8, 1.6% SDS, 10% Glycerol, 10% DTT, 0.01% bromophenol blue) and transferred to a new tube containing Avidin as a stabilizer (0.11 μg/μl, Sigma). Samples were resolved by polyacrylamide gel electrophoresis and protein bands were visualized either by silver staining or by Western blotting using anti-FLAG to detect SMARCAL1 and ZRANB3, and by anti-His primary antibodies to detect RAD51C of the BCDX2 complex by standard procedures.

### Antibodies

The antibodies used for Western blotting, immunoprecipitation and DNA fiber assay were used as follows: Mouse anti-RAD51B (Santa Cruz sc-377192; 1:1000 dilution for WB), Mouse anti-XRCC2 (Santa Cruz sc-365854; 1:1000 dilution for WB), Rabbit anti-RAD51C (Abcam ab95069; 1:1000 dilution for WB), Rabbit anti-RAD51D (Abcam ab202063; 1:1000 dilution for WB), Rat anti-BrdU (Abcam ab6326; 1:100 dilution for DNA fiber assay), Mouse anti-BrdU (Becton Dickinson BD347580; 1:100 dilution for DNA fiber assay), Mouse anti-SMARCAL1 (Santa Cruz sc-376377; 1:1000 dilution for WB), Mouse anti-BRCA1 (Santa Cruz sc-6954; 1:100 dilution for WB), Rat anti-TUBULIN (Abcam ab-6160; 1:50 000 dilution for WB), Goat anti-mouse Alexa Fluor 488 (Thermo Fisher A-11029; 1:300 dilution for DNA fiber assay), Goat anti-rabbit Alexa Fluor 594 (Thermo Fisher A-11008; 1:300 dilution for DNA fiber assay), Mouse anti‐his (MBL D291‐3; 1 μg for pulldown assay), Mouse anti‐FLAG (Sigma F3165; 1:2,000 dilution for WB), Mouse anti-MBP (MBL M091-3; 1:1000 dilution for WB), Rabbit anti-RAD51 (Abcam ab133534; 1:1000 dilution for WB).

### Cellular assays

MCF10A cells were maintained in a 1:1 mixture of DMEM and Ham's F12 medium (Thermo Fisher Scientific), supplemented with 5% horse serum (Thermo Fisher Scientific), 20 ng/ml human epidermal growth factor (Peprotech), 100 ng/ml cholera toxin, 10 μg/ml insulin and 0.5 μg/ml hydrocortisone (Sigma-Aldrich). The human embryonic kidney fibroblast cell line HEK293T was maintained in DMEM supplemented with 10% Fetalgro bovine growth serum. Gateway LR recombination (Thermo Fisher Scientific) was used to recombine pDONR223-SMARCAL1(F446A) with the lentiviral expression vector pHAGE-C-FLAG-HA-DEST (39). Recombinant lentiviruses were generated by cotransfecting helper packaging vectors together with lentiviral vectors into HEK293T cells using the TransIT-293 transfection reagent (Mirus Bio). Virus-containing supernatants were collected 48 h after transfection and utilized to infect MCF10A cells in the presence of 8 μg/ml polybrene. 48 h after viral addition, MCF10A cells were selected using 1 μg/ml puromycin for 3 days. To perform RNAi treatments, MCF10A SMARCAL1 KO cells complemented with WT and F446A mutant SMARCAL1 were transfected with control or BRCA1 siRNA (GE Dharmacon) using lipofectamine RNAiMAX (Thermo Fisher Scientific) according to manufacturer's instructions and subjected to DNA fiber assays 3 days after transfection. To analyze cell lysates by Western blotting, cells were collected by trypsinization and lysed in SB lysis buffer (62.5 mM Tris–HCl pH 6.8, 1.25% SDS, 12% glycerol, 0.71 M [5%] β-ME, 0.002% bromophenol blue). Whole cell extracts were sonicated and heated for 5 min at 95°C. Following gel electrophoresis and transfer of cell extracts onto nitrocellulose, membranes were incubated for 1 h or overnight in blocking buffer (5% milk in TBS + 0.1% Tween20). Membranes were subsequently incubated with primary antibodies diluted in antibody blocking buffer for 2 h at room temperature or overnight at 4°C. Detection was achieved using appropriate horseradish peroxidase (HRP)-conjugated secondary antibodies. Anti-SMARCAL1 (1:1000, Santa Cruz Biotechnology), anti-BRCA1 (1:100, Santa Cruz Biotechnology) and anti-TUBULIN (1:50000, Abcam) antibodies were used in western blot experiments.

### Single-molecule analysis of DNA replication

Exponentially growing MCF10A cells were pulse-labeled with 30 μM CldU (25 min), washed and exposed to 150 μM IdU (35 min). After exposure to the second nucleotide analog, the cells were washed again in warm 1× PBS and treated or not for 4 h with hydroxyurea (HU, 2 mM, Sigma). Labeled cells were trypsinized and resuspended in ice-cold PBS at 4 × 10^5^ cells/ml. Two microliters of this suspension were spotted onto a pre-cleaned glass slide and lysed with 10 μl of spreading buffer (0.5% SDS in 200 mM Tris–HCl pH 7.4, 50 mM EDTA). After 6 min, the slides were tilted at 15° relative to horizontal, allowing the DNA to spread. Slides were air-dried, fixed in methanol and acetic acid (3:1) for 2 min, rehydrated in PBS for 10 min and denatured with 2.5 M HCl for 50 min at room temperature. Slides were then rinsed in PBS and blocked in PBS + 0.1% Triton X-100 (PBS-T) + 5% BSA for 1 h at room temperature. Rat anti-BrdU (1:100, Abcam) and mouse anti-BrdU (1:100, BD) were then applied to detect CldU and IdU, respectively. After 1 h incubation, the slides were washed in PBS and stained with Alexa Fluor 488-labeled goat anti-mouse IgG1 antibody and Alexa Fluor 594-labeled goat anti-rat antibody (1:300 each, Thermo Fisher Scientific). Slides were mounted in Prolong Gold Antifade (Thermo Fisher Scientific) and held at –20°C. Replication tracks were imaged on a Nikon Eclipse 90i microscope fitted with a PL Apo 40×/0.95 numerical aperture (NA) objective and measured using ImageJ software. In each experiment, 100 or more dual-labeled tracts were measured for fork degradation estimation.

## RESULTS

### SMARCAL1, ZRANB3 and HLTF have unequal biochemical activities

**Figure 1.**
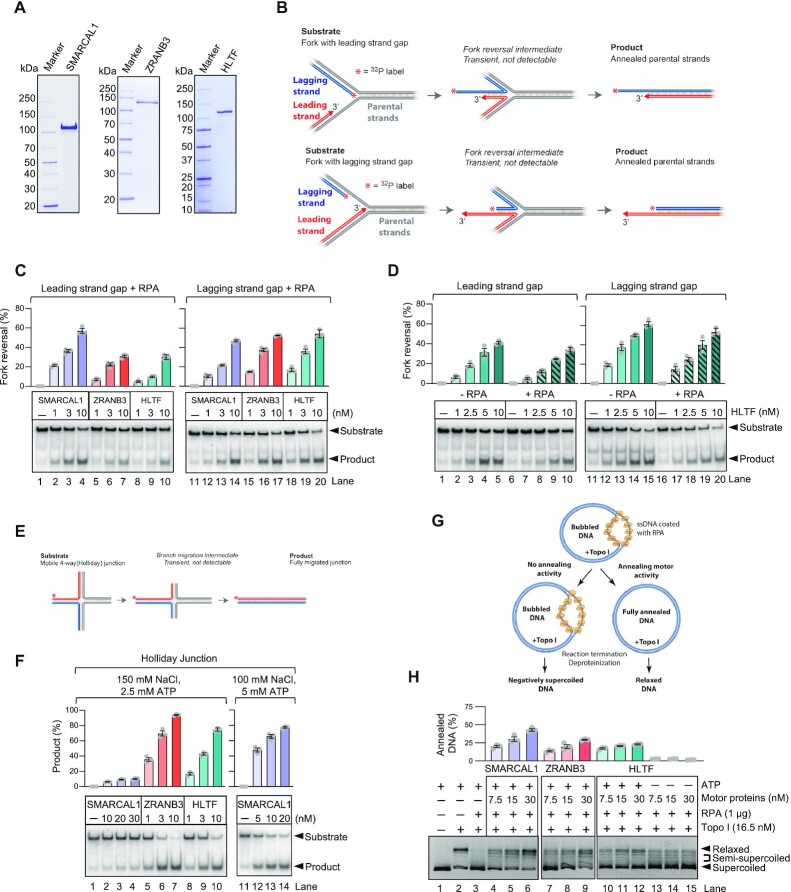
SMARCAL1, ZRANB3 and HLTF possess distinct biochemical activities. (**A**) Recombinant SMARCAL1, ZRANB3, and HLTF were analyzed by polyacrylamide gel electrophoresis and stained with Coomassie Brilliant Blue. (**B**) A schematic of replication fork reversal assay (leading and lagging strand gap structure is shown). (**C**) Fork reversal assays with SMARCAL1, ZRANB3 and HLTF with RPA (3 nM). Top, quantifications (error bars show SEM of three replicates); bottom, representative experiments. (**D**) Fork reversal assays with HLTF without or with RPA (3 nM). Top, quantifications (error bars indicate SEM of three replicates); bottom, representative experiments. (**E**) A schematic of Holliday junction branch migration assay. (**F**) Holliday junction branch migrations assay with SMARCAL1, ZRANB3 and HLTF. Top, quantifications (error bars indicate SEM of three replicates); bottom, representative experiments. (**G**) A schematic of topoisomerase-coupled annealing assay. (**H**) Comparison of SMARCAL1, ZRANB3 and HLTF in topoisomerase-coupled annealing assays. ATP hydrolysis by HLTF is required, as no detectable annealing was observed without ATP. Top, quantifications (error bars indicate SEM of three replicates); bottom, representative experiments.

SMARCAL1, ZRANB3 and HLTF have all been implicated in replication fork reversal *in vitro* and *in vivo* (15,23,24,40–43). The loss of either of these enzymes was shown to abolish nascent DNA degradation in BRCA1/2-deficient cells, suggesting that these factors may act in a non-redundant manner to promote fork reversal (12,23). We expressed and purified SMARCAL1, ZRANB3 and HLTF from insect *Sf*9 cells to be able to analyze their biochemical activities side by side (Figure 1A). Our preparations of SMARCAL1, ZRANB3 and HLTF were largely monomeric, with a small fraction of dimers in case of ZRANB3 and HLTF, as determined by mass photometry (Supplementary Figure S1A). All three translocases hydrolyzed ATP, as expected, with SMARCAL1 showing the highest specific activity, followed by HLTF and ZRANB3 (Supplementary Figure S1B). We next set out to compare the relative activities of these motor proteins in biochemical assays mimicking elements of fork reversal. We first used oligonucleotide-based DNA substrates resembling stalled replication forks with ssDNA gaps either in the leading or the lagging DNA strand (Figure 1B). We observed, as reported previously, that SMARCAL1 in the presence of the ssDNA binding protein RPA was more efficient on forks with leading strand gaps, as opposed to ZRANB3, which prefers RPA on lagging strand gaps (37) (Figure 1C). Using the leading strand gap substrate, SMARCAL1 was also more efficient than HLTF (Figure 1C), while the three translocases exhibited similar specific activities on the lagging strand gap substrate (Figure 1C). In contrast to the activities of SMARCAL1 and ZRANB3 that are regulated by RPA (37), the function of HLTF was not RPA sensitive (Figure 1D), in agreement with previous data (44). During fork reversal, the initial annealing of the nascent DNA strands leads to the formation of a 4-way junction (Holliday junction, HJ), which is further branch migrated by the motor proteins, leading to reversed forks of up to several kilobases in length (9). Using a mobile HJ substrate to assay for branch migration (Figure 1E), we observed that SMARCAL1 was in contrast the least efficient enzyme, essentially incapable of branch migration at physiological (150 mM) salt concentrations. However, under less restrictive conditions in lower salt (100 mM) and with increased concentration of ATP, the branch migration activity of SMARCAL1 was readily detected (Figure 1F, right panel). Instead, both ZRANB3 and HLTF were highly and comparably efficient in branch migration at 150 mM salt (Figure 1E, F). The nearly complete disruption of fork reversal upon depletion of either SMARCAL1, ZRANB3 or HLTF, as observed in cellular experiments (12,23), might suggest that these enzymes function together at the same time. We performed experiments with different combinations of SMARCAL1, ZRANB3 or HLTF, and only observed additive effects (Supplementary Figure S1 C,D). The absence of synergy, at least in the reconstituted system, rather argues against a joint function.

The activity of the SMARCAL1 and ZRANB3 enzymes was first analyzed by a topoisomerase-coupled assay that monitors the annealing of RPA-coated ssDNA bubbles in plasmid DNA, which can be observed as changes in DNA topology (18,38,45). Such activity is thought to mimic the initial stages of fork remodeling. Both SMARCAL1 and ZRANB3 were shown to anneal the RPA-coated DNA bubbles as a result of their motor functions, as ATP hydrolysis is required for this reaction (18,38,45). However, the specific activities of SMARCAL1 and ZRANB3 have not been directly compared. We observed that SMARCAL1 was comparably efficient to ZRANB3, while HLTF showed much lesser capacity to anneal DNA in this assay (Figure 1G, H).

Taken together, our data support and extend previous observation that SMARCAL1, ZRANB3 and HLTF possess quite different biochemical activities and substrate preferences. Our results are in agreement with models positing that fork reversal is not catalyzed by a single enzyme in a processive manner, but that it is rather a dynamic process that involves the sequential engagement of several factors (9,23,46,47). SMARCAL1 is efficient at DNA annealing, followed by ZRANB3, while HLTF and ZRANB3 are more efficient in branch migration.

### SMARCAL1 specifically anneals RPA-coated ssDNA

**Figure 2.**
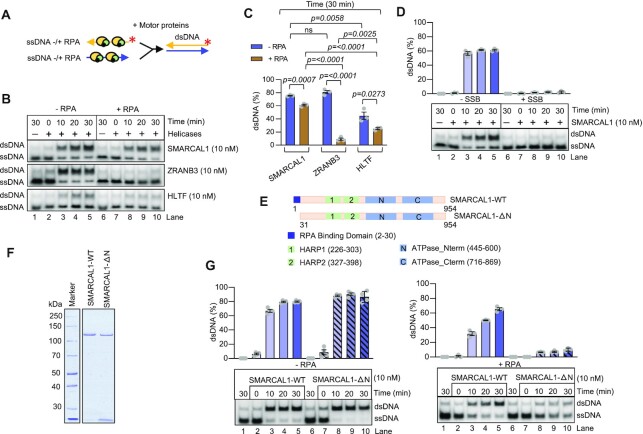
SMARCAL1 anneals RPA-coated ssDNA. (**A**) A schematic of ssDNA annealing assay. (**B**) Annealing of ssDNA by SMARCAL1, ZRANB3 and HLTF without or with RPA (4 nM). Representative experiments are shown. (**C**) Quantification of experiment as in (**B**) at 30 min (error bars indicate SEM of three replicates). Two-tailed unpaired t-test was performed to generate the *P* values. (**D**) Annealing of ssDNA by SMARCAL1 without or with human mitochondrial SSB (4 nM). Top, quantifications (error bars indicate SEM of three replicates); bottom, representative experiment. (**E**) Top, a schematic showing domain organization of SMARCAL1. RPA binding domain is located in the N-terminal part of SMARCAL1 (indicated in dark blue). SMARCAL1-ΔN lacking RPA binding domain is shown below. (**F**) Recombinant SMARCAL1-WT and SMARCAL1-ΔN were analyzed by polyacrylamide gel electrophoresis and stained with Coomassie Brilliant Blue. (**G**) A comparison of SMARCAL1-WT and SMARCAL1-ΔN in ssDNA annealing without or with RPA (4 nM). Top, quantifications (error bars indicate SEM of three replicates); bottom, representative experiments.

Several helicases, such as members of the RecQ family, were reported to anneal two ssDNA molecules, but the reactions were inhibited by RPA (48,49). Considering that cellular RPA concentration is thought to be sufficient to coat all ssDNA in most cases, the physiological relevance of these observations remains unclear. In this regard, the reported activities of the RecQ family members differ from the canonical RecO/RAD52 family annealing proteins, which anneal RPA-coated ssDNA to promote homologous recombination and single strand annealing (SSA) (50,51). Similarly, HELQ was recently described to anneal RPA-coated ssDNA (52). We next set out to investigate whether the fork reversal enzymes can anneal two ssDNA molecules similarly as RAD52 or HELQ. The annealing of the bubbled DNA in the topoisomerase-coupled assays could result from an annealing activity *per se*, or can be a consequence of the dsDNA translocase activity rezipping the bubble from the side. To distinguish between these two possibilities, we set to define the function of the fork remodelers in complementary ssDNA annealing (Figure 2A). We observed that SMARCAL1, ZRANB3 and HLTF were all able to anneal free ssDNA, which can be explained by multiple ssDNA binding sites on a single enzyme or by enzyme oligomerization, which can bring multiple ssDNA molecules to close proximity, stimulating their annealing (Figure 2B, C). However, in the presence of RPA, the ssDNA annealing by ZRANB3 and HLTF was strongly reduced, similarly as observed with the RecQ family helicases (48), arguing against physiological relevance. In contrast, ssDNA annealing by SMARCAL1 remained highly proficient in the presence of RPA (Figure 2B, C). Unlike with RPA, the ssDNA annealing capacity of SMARCAL1 was abrogated when the ssDNA was pre-coated with mitochondrial SSB, showing that the annealing of ssDNA by SMARCAL1 is allowed in the presence of RPA in a specific manner (Figure 2D). The annealing activity of SMARCAL1 was also observed in the absence of ATP, or when using the motor-dead SMARCAL1 (D549A, E550A, SMARCAL1-HD) variant showing that this particular activity is not ATPase dependent (Supplementary Figure S2A), as is the case of RAD52 (51), but distinct from HELQ (52). Therefore, the annealing of two complementary ssDNA molecules by SMARCAL1 mechanistically differs from the annealing of bubbled DNA in the topoisomerase coupled assays, which largely require ATP hydrolysis, and hence likely results from the enzyme translocating on dsDNA (18,38,45).

The N-terminal region of SMARCAL1 contains a previously defined RPA-binding site, the integrity of which is required for the recruitment of SMARCAL1 to DNA damage sites, and to direct SMARCAL1 to substrates with RPA-coated ssDNA gaps (14) (Figure 2E). However, SMARCAL1-ΔN, lacking the RPA interaction domain, is still proficient in the bubbled DNA annealing assay (18,53). To test for the requirement for direct interaction between SMARCAL1 and RPA in ssDNA annealing, we expressed and purified the SMARCAL1-ΔN variant (Figure 2F). The truncated SMARCAL1 was fully proficient in DNA branch migration in the absence of RPA and identical to wild type SMARCAL1 as an ATPase (Supplementary Figure S2B, C). However, the mutant was inefficient in ssDNA annealing in the presence of RPA, showing that the direct interaction of SMARCAL1 with RPA is essential for this activity (Figure 2G). Our results reveal that SMARCAL1 possesses a strand annealing activity similar to members of the RAD52 protein family, which also rely on specific interaction with RPA (51). We suggest that such activity may be employed during the very initial steps of fork reversal, when the daughter ssDNA molecules are separated from the parental strands and need to anneal with each other. These results further underline the mechanistic differences between the fork remodeling enzymes SMARCAL1, ZRANB3 and HLTF.

### RAD51 and BCDX2 paralogs promote motor-driven strand annealing activity of SMARCAL1 and ZRANB3 but not HLTF

**Figure 3.**
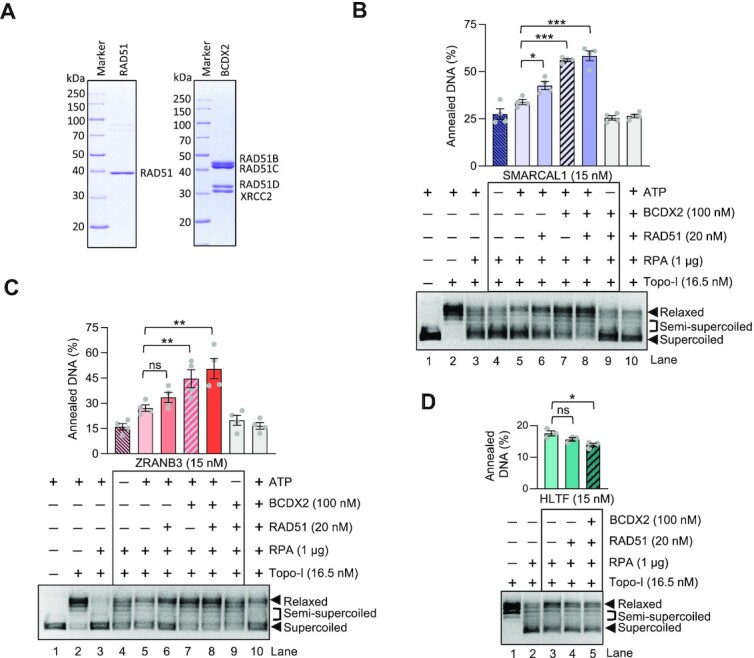
RAD51 and BCDX2 promote SMARCAL1 and ZRANB3 mediated bubbled DNA annealing. (**A**) Recombinant RAD51 and BCDX2 were analyzed by polyacrylamide gel electrophoresis and stained with Coomassie Brilliant Blue. (**B**) Annealing helicase assay. RAD51 and BCDX2 separately stimulate SMARCAL1-mediated annealing of RPA-coated ssDNA. Top, quantifications (error bars indicate SEM of four replicates); bottom, representative experiment. Statistical significance; *(*P* < 0.05), ***(*P* < 0.001), two-tailed *t*-test. (**C**) RAD51 and BCDX2 separately promote ZRANB3 mediated strand annealing. Top, quantifications (error bars indicate SEM of four replicates); bottom, representative experiment. Statistical significance bars; ns (*P* > 0.05, not significant); **(*P* < 0.01); two-tailed *t*-test. (**D**) RAD51 and BCDX2 do not promote HLTF-mediated DNA annealing. Top, quantifications (error bars indicate SEM of three replicates); bottom, representative experiment. Statistical significance; ns (*P* > 0.05, not significant); *(*P* < 0.05), two-tailed *t*-test.

Challenged DNA replication forks may undergo reversal, and reversed replication forks must be subsequently protected by RAD51 to prevent pathological nascent DNA degradation (2,3,8,9,54). However, RAD51, along with the RAD51 paralog BCDX2 complex, were also paradoxically implicated in promoting fork reversal, through a yet unknown mechanism (10,22). To elucidate whether the function of RAD51 and the RAD51 paralogs in fork remodeling may be direct, we next expressed and purified RAD51 and the BCDX2 complex (Figure 3A and Supplementary Figure S3A). The BCDX2 complex was obtained upon co-expression of all subunits in insect cells, as the preparation of the individual proteins resulted in poor yields and solubility. The BCDX2 complex did not aggregate, bound ssDNA and very weakly hydrolyzed ATP, as observed previously (Supplementary Figure S3B–D) (55). Using a mass photometer, the stoichiometry of the obtained complex was consistent with that of a dimer of the heterotetramer (Supplementary Figure S3E).

We next set out to test whether RAD51 and the BCDX2 complex affect the strand annealing and motor activities of SMARCAL1. To this point, we used the established topoisomerase-coupled assay (18,38,45). Strikingly, we observed that low concentrations of RAD51 and the BCDX2 complex promoted bubbled DNA annealing by SMARCAL1, while none of these co-factors had a notable capacity to mediate DNA annealing *per se* without SMARCAL1, even at much higher concentrations (Figure 3B, Supplementary Figure S3F, G). Additionally, controls where no ATP was used or helicase-dead SMARCAL1 variant replaced the wild type protein largely abolished DNA annealing, indicating that a large proportion of the relaxed DNA signal in the assay can be linked to the ATP hydrolysis-driven translocation activity of SMARCAL1 (Figure 3B, Supplementary Figure S3H). Similarly to SMARCAL1, we observed that RAD51 and the BCDX2 paralogs promoted the annealing capacity of ZRANB3. As above with SMARCAL1, we observed that RAD51 and BCDX2 could both promote ZRANB3 independently of each other (Figure 3C). Only limited changes in DNA topology were observed when using helicase-dead ZRANB3 and all co-factors, demonstrating that the majority of the signal in the assay can be linked to the motor activity of ZRANB3 leading to DNA annealing (Supplementary Figure S3I). Differently from SMARCAL1 and ZRANB3, HLTF was not stimulated by either RAD51 or BCDX2 (Figure 3D). Together, we show that RAD51 and BCDX2 promote the translocation-driven annealing of RPA-coated bubbled DNA by SMARCAL1 and ZRANB3, suggesting that the co-factors may have a direct role in fork reversal to stimulate the DNA translocases. We note that the motor activity of HELQ was recently described to be stimulated by RAD51 (52), so a structural function of RAD51 in regulating DNA translocases may be a more common mechanism.

### SMARCAL1 and ZRANB3 physically interact with RAD51 and BCDX2

**Figure 4.**
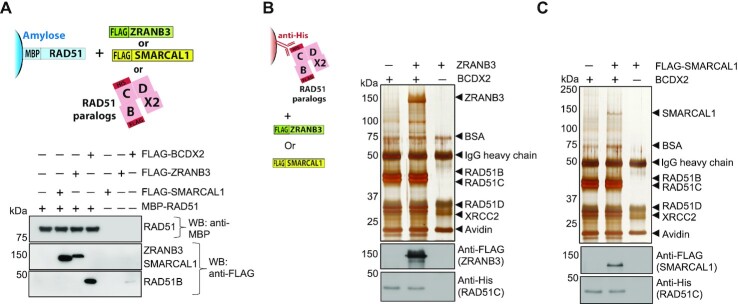
SMARCAL1 and ZRANB3 physically interact with RAD51 and BCDX2. (**A**) Soluble extract from *E. coli* containing MBP-RAD51 (bait) was immobilized on amylose resin and incubated with purified recombinant proteins (RAD51 paralogs BCDX2, ZRANB3 or SMARCAL1, [prey]) as indicated. Western blot analyses were performed with anti-MBP and anti-FLAG antibodies. (**B**) and (**C**) Anti-His antibody was coupled to Protein G agarose, bound to the BCDX2 complex (bait) and tested for interaction with ZRANB3 (prey) or SMARCAL1 (prey), respectively. Samples were subjected to either silver staining or Western blot analysis with anti-FLAG and anti-His antibodies.

To test whether the functional interplay between SMARCAL1 and ZRANB3 with RAD51 and BCDX2 may involve direct physical interactions, we immobilized RAD51 and performed pulldown experiments with the co-factors. We observed that BCDX2 interacted with RAD51 (the RAD51B component was detected), as described previously (56). Importantly, we found that both SMARCAL1 and ZRANB3 also interacted with RAD51 (Figure 4A). We next immobilized the BCDX2 complex, and observed reciprocally a direct interaction with SMARCAL1 and ZRANB3, as detected by Western blotting and silver staining (Figure 4B, C). These results collectively suggest that the interplay of SMARCAL1 and ZRANB3 with RAD51 and BCDX2 likely involves direct physical interactions.

### Point mutation in SMARCAL1 disrupts physical and functional interactions with RAD51

**Figure 5.**
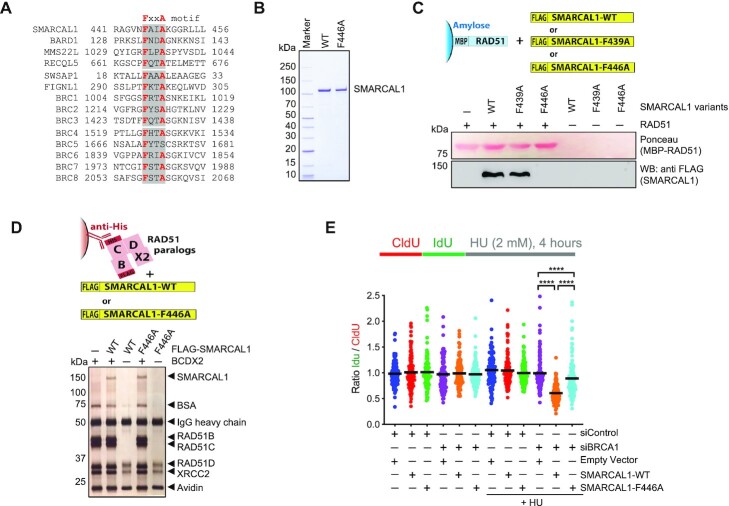
Identification and functional characterization of RAD51 interaction site in SMARCAL1. (**A**) Multiple sequence alignment showing the presence of a consensus FxxA motif in SMARCAL1 along with previously characterized RAD51 interacting proteins or BRCA2 domains (highlighted in grey with bold red letters). (**B**) Recombinant SMARCAL1-WT and SMARCAL1-F446A were analyzed by polyacrylamide gel electrophoresis and stained with Coomassie Brilliant Blue. (**C**) SMARCAL1-F446A fails to interact with RAD51. Soluble extract from *E. coli* containing MBP-RAD51 (bait) was immobilized on amylose resin and incubated with purified recombinant SMARCAL1 variants (prey). Ponceau staining shows RAD51. Western blot analysis was performed with anti-FLAG antibody to detect SMARCAL1. (**D**) SMARCAL1-F446A, as SMARCAL1-WT, interacts with the BCDX2 complex. Anti-His antibody was immobilized on protein G agarose, bound to BCDX2 complex (bait) and tested for interaction with SMARCAL1 variants (prey). Samples were subjected to silver staining. (**E**) DNA fiber assay to monitor SMARCAL1-mediated nascent DNA degradation in BRCA1-deficient cells. Wild type or SMARCAL1-F446A proteins were expressed in SMARCAL1 KO MCF10A cells upon BRCA1 depletion, as indicated. SMARCAL1-deficiency renders BRCA1-depleted cells resistant to replication fork degradation upon hydroxyurea (HU) treatment, as a result of impaired fork reversal. Top: a schematic of the assay: CldU (25 min), IdU (35 min) pulse-labeling protocol to evaluate fork degradation upon HU treatment. Under wild type condition the ratio of IdU/CldU tract length will remain ∼1, however if there is fork degradation this ratio will be <1. Bottom: graphical representation of IdU/CIdU tract length ratio. The median value of 100 or more IdU and CldU tracts per experimental condition is indicated. Statistical analysis was conducted using Mann–Whitney test (****P < 0.0001). Data are representative of two independent experiments.

We next set out to define motifs in SMARCAL1 and ZRANB3 that mediate the interactions with RAD51 and BCDX2. We failed to identify an interaction motif with BCDX2, but we found regions in SMARCAL1 mediating the binding to RAD51. Physical and functional interactions between RAD51 and many of its co-factors, such as BRCA2, BARD1, MMS22L, RECQL5, SWSAP1 and FINGL1 are mediated by the FXXA motif (32,57–61). We identified such a motif in SMARCAL1, which is conserved in evolution (Figure 5A, Supplementary Figure S4A). The FXXA motif is positioned ahead of the conserved αI SNF2 family ATPase domain (Supplementary Figure S4B). The mutation of phenylalanine 446 into alanine (F446A) in SMARCAL1 disrupted the physical interaction with RAD51 (Figure 5B, C). In contrast, disruption of F439, which is part of a less conserved FXXA sequence in human SMARCAL1 upstream of F446, did not impair the interaction (Figure 5C, Supplementary Figure S4A). We note that SMARCAL1-F446A variant *per se* was very similar to wild type SMARCAL1 in its fork reversal and ATPase capacities *in vitro* and retained its physical interaction with the BCDX2 complex (Supplementary Figure S4C, D and Figure 5D). ZRANB3 contains a phenylalanine at the analogous position to SMARCAL1 ahead of the ATPase domain. The phenylalanine however does not conform to the FXXA motif, and the F47A substitution mutant retained its capacity to interact with RAD51 and was impaired in its ATPase activities (Supplementary Figure S4E-H). We next found that mutation F736A in ZRANB3 disrupted interaction with RAD51, however it is likely that the mutation affected the fold of the substrate recognition domain, as it likewise abolished the biochemical activities of ZRANB3 and may thus not represent a direct interaction motif (Supplementary Figure S4I–K) (62). Due to the impact of this mutation on the activities of ZRANB3 *per se*, we could not test for the physiological relevance of the interaction with RAD51.

To investigate the physiological relevance of the disrupted physical interaction between SMARCAL1 and RAD51, we used MCF10A SMARCAL1 KO cells (23), which were complemented with either wild type SMARCAL1 or the F446A variant (Figure 5E). Following replication stress induced by hydroxyurea, it was previously demonstrated that SMARCAL1-mediated fork reversal can lead to nascent DNA degradation, as long as the nascent DNA is not protected by RAD51 (23). The nascent DNA degradation is evident in cells lacking BRCA1 or BRCA2, which may be required to recruit, load or stabilize RAD51. In agreement with previous data (23), we observed extensive nascent DNA degradation in BRCA1-depleted SMARCAL1 KO cells reconstituted with wild type SMARCAL1 (Figure 5E). In contrast, such extensive DNA degradation was not observed in BRCA1-depleted SMARCAL1 KO cells reconstituted with empty vector (no SMARCAL1), and it was partially attenuated in cells with SMARCAL1-F446A, which was expressed at levels comparable to wild type (Supplementary Figure S4L). Taking into consideration that nascent DNA degradation in BRCA-deficient cells requires the fork reversal activity of SMARCAL1 (23), our results suggest that SMARCAL1-F446A, which does not interact with RAD51, might display a defective fork reversal activity in mammalian cells.

## DISCUSSION

Here, we used biochemistry to study the function of the replication fork remodelers SMARCAL1, ZRANB3 and HLTF, and their regulation by RAD51 and RAD51 paralogs.

### Novel strand annealing function of SMARCAL1

Depletion of either SMARCAL1, ZRANB3 or HLTF brings about a profound defect in replication fork reversal, as observed by electron microscopy, or by proxy methods scoring for e.g. nascent DNA degradation in various genetic backgrounds upon stress (12,15,40–42,63). To better define the function of the fork remodelers, we compared their specific activities on various substrates mimicking elements of fork reversal. We found that SMARCAL1, but not ZRANB3 or HLTF, has a unique capacity to anneal RPA-coated ssDNA, a function reminiscent of the RAD52 protein family or as recently described for HELQ (52). The annealing function of SMARCAL1 depends on the RPA interaction motif within the N-terminus of SMARCAL1. The RPA interaction motif was earlier found to be necessary for the recruitment of SMARCAL1 to DNA damage sites and for its physiological function in cells (14). We hypothesize that such annealing function might be relevant during the initial annealing of the displaced daughter strands during the early steps of fork reversal. The annealing activity of SMARCAL1, similarly to RAD52, does not involve ATPase activity.

Previously, the function of SMARCAL1 and ZRANB3 was monitored in assays scoring for the annealing of bubbled DNA within circular plasmid (18,38,45). Such activity, in contrast to annealing of RPA-coated ssDNA oligonucleotides, is dependent on the motor activities of the remodelers. In case of SMARCAL1, its direct interaction with RPA is not required (18,53). We show that in contrast to SMARCAL1 and ZRANB3, which are similarly proficient in the bubbled DNA rezipping, HLTF was largely inefficient in this assay. Branch migration follows the initial strand annealing during fork reversal. Using 4-way junction substrates to monitor branch migration, HLTF and ZRANB3 were the most active enzymes, while SMARCAL1 was the least efficient. Our experiments demonstrated that the fork remodelers possess quite different specific activities with respect to the substrates used, which extends results reported previously (18,23,47,63). The data support a model where fork remodeling is not catalyzed by a single enzyme in a processive manner, but it is rather a process with various remodelers acting in a distributive fashion (63), depending on the nature of the DNA intermediate and the substrate preference of the respective remodeler. Such model would explain the non-redundant relationship of the remodelers in fork reversal (23,47,63).

### RAD51 and the paralog complex BCDX2 directly promote SMARCAL1 and ZRANB3

Previous cellular data suggested that RAD51 and the BCDX2 complex promote fork reversal, but the underlying mechanism was not clear (10,22). The function of RAD51 in fork remodeling was shown to be genetically separable and thus different from its canonical role in homologous recombination (20). Specifically, the strand exchange function of RAD51 was dispensable, pointing at a potential non-catalytic function (20). We show here that RAD51 and the RAD51 BCDX2 paralog complex stimulate the strand annealing and branch migration activities of SMARCAL1 and ZRANB3, two of the key enzymes implicated in fork reversal. SMARCAL1 and ZRANB3 were stimulated when RAD51 concentration was too low to support a nucleoprotein filament formation. Recently, RAD51 was shown to promote the helicase activity of HELQ (52), showing that RAD51 may structurally promote several DNA motor proteins.

In accord with a recent cellular study that identified a function of BCDX2 in promoting fork reversal (22), we find that the paralog complex also directly stimulates SMARCAL1 and ZRANB3. Unexpectedly, RAD51 and BCDX2 stimulated SMARCAL1 and ZRANB3 independently of each other, as we observed mostly additive effects when combined. The function of the RAD51 paralogs, such as the BCDX2 complex, in homologous recombination remains poorly defined. Some reports suggest a joint function for the paralogs and RAD51. Specifically, BCDX2 was shown to have a classical recombination mediator function to load RAD51 on RPA-coated ssDNA (64–66), to remodel RAD51 filaments for activation (67), or to make them more resistant against disruption (68). However, RAD51-independent function of the RAD51 paralogs were also identified in cellular studies, such as in the single-strand annealing pathway of DSB repair (69), and BCDX2 was also found to physically and functionally associate with the HELQ helicase (70). The function of BCDX2 to promote SMARCAL1/ZRANB3 described here *in vitro* also does not require RAD51.

### The interplay of RAD51 and paralogs in promoting SMARCAL1 and ZRANB3 involves physical interactions

RAD51 and BCDX2 did not stimulate HLTF, a third enzyme shown to catalyze fork reversal, suggesting a specificity in the interplay of SMARCAL1 and ZRANB3 with RAD51 and BCDX2. In accord, we found that RAD51 and BCDX2 physically interact with SMARCAL1 and ZRANB3. We could then map the RAD51 interaction site in SMARCAL1 and constructed a single point mutant (SMARCAL1-F446A) that disrupted the physical interaction with RAD51. The SMARCAL1 mutant was not impaired in its activities *per se*, but was deficient in promoting nascent DNA degradation in BRCA1-deficient cells, a process that requires the fork reversal activity of SMARCAL1, supporting the idea that the identified interplay of SMARCAL1 and RAD51 is physiologically relevant.

## DATA AVAILABILITY

Primary data are available in the manuscript or as Supplementary Data.

## Supplementary Material

gkac583_Supplemental_Files
