# Supplementary material for: Strand annealing and motor driven activities of SMARCAL1 and ZRANB3 are stimulated by RAD51 and the paralog complex
Source: Nucleic Acids Res. 2022 Jul 8;50(14):8008–22. doi: 10.1093/nar/gkac583 (PMC9371921; doi:10.1093/nar/gkac583)
Supplement: gkac583_Supplemental_Files [file gkac583_supplemental_files.zip › Halder et al., supplementary information.pdf]

Supplementary data

Strand annealing and motor driven activities of SMARCAL1 and ZRANB3 are stimulated by RAD51 and the paralog complex

Swagata Halder, Lepakshi Ranjha, Angelo Taglialatela, Alberto Ciccia and Petr Cejka

Summary:

4 supplementary figures

1 supplementary table

Supplementary Figure 1

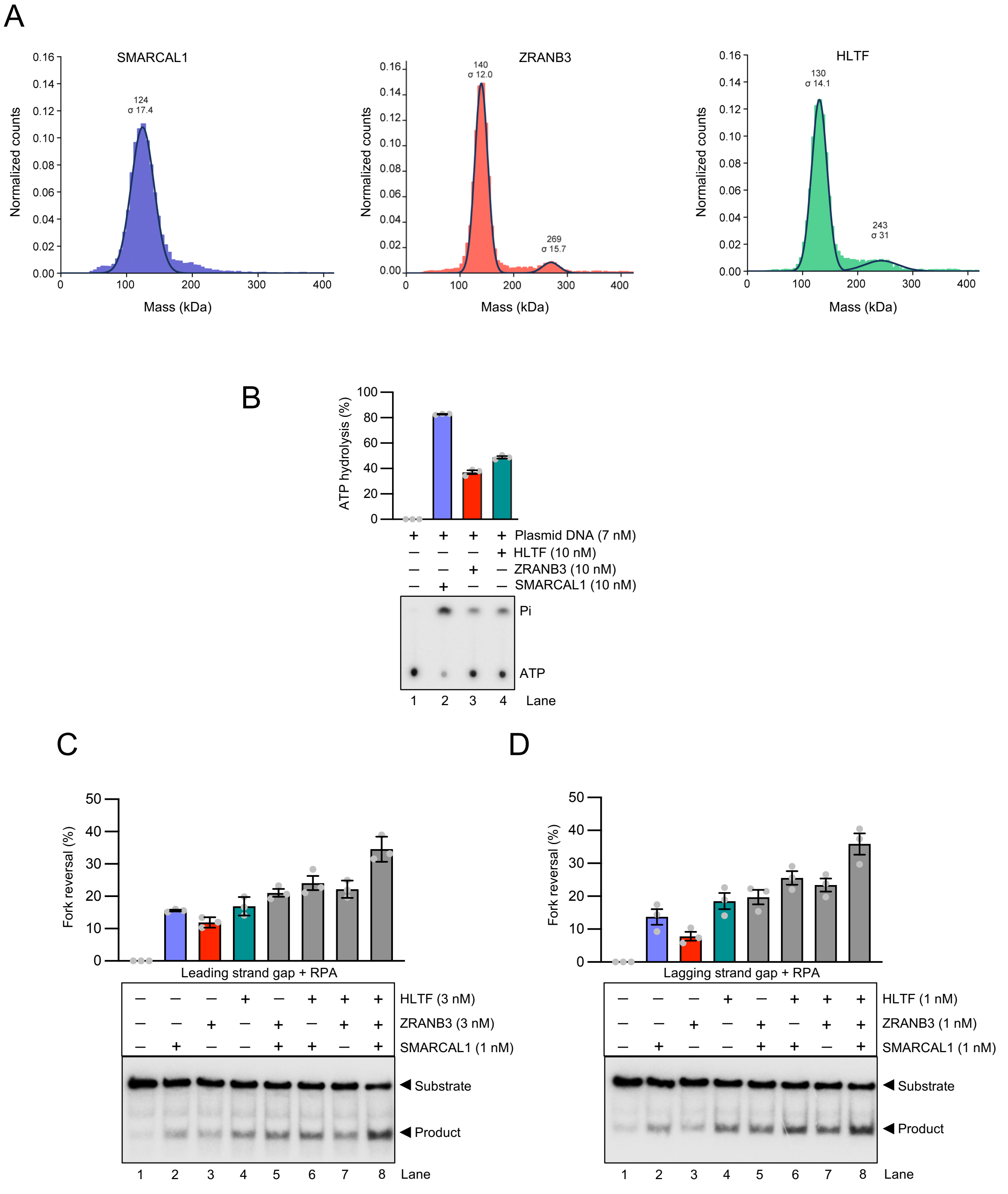

Supplementary Figure 1 (Related to Figure 1)

(A) Mass-photometry analysis of SMARCAL1, ZRANB3 and HLTF suggesting they are mostly monomers. Expected molecular weight of SMARCAL1, ZRANB3 and HLTF is 110 kDa, 123 kDa and 116 kDa, respectively.

(B) A comparison of ATP hydrolysis by SMARCAL1, ZRANB3 and HLTF. Top, quantifications (error bars indicate SEM of three replicates); bottom, a representative experiment.

(C) and (D) Analysis of SMARCAL1, ZRANB3 and HLTF single or combined in fork reversal assays using either leading strand (C) or lagging strand (D) gap substrates. Top, quantifications (error bars indicate SEM of three replicates); bottom, representative experiment.

Supplementary Figure 2

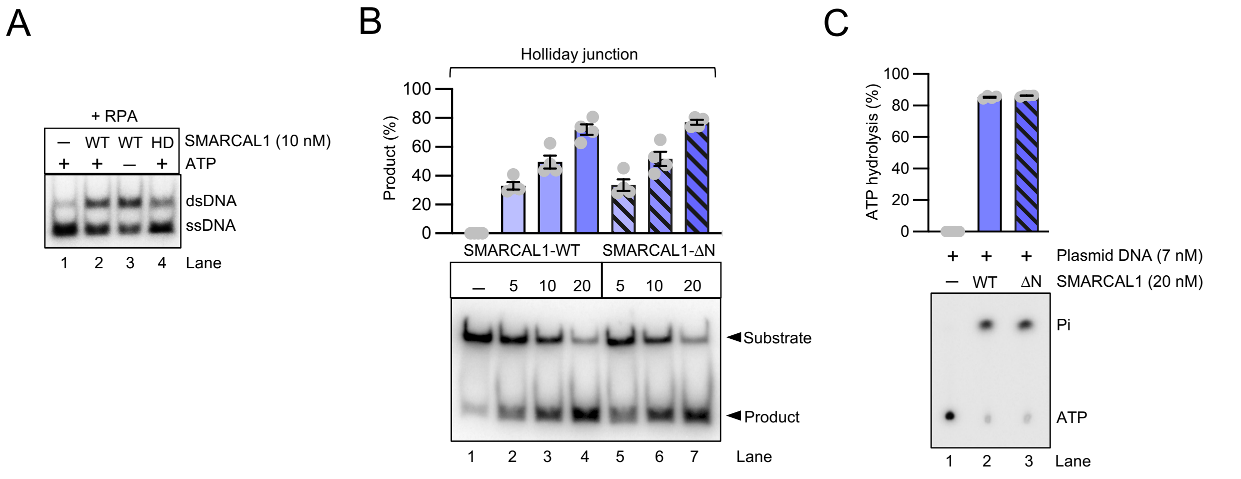

Supplementary Figure 2 (Related to Figure 2)

(A) Annealing of ssDNA by SMARCAL1 wild type or helicase-dead (D549A, E550A, SMARCAL1-HD) in the presence or absence of ATP.

(B) Holliday junction branch migration by SMARCAL1-WT (residues 1-954) and SMARCAL1-ΔN (residues 31-954). Experiment was performed with 100 mM NaCl and 5 mM ATP. Top, quantifications (error bars indicate SEM of four replicates); bottom, representative experiment.

(C) ATP hydrolytic capacity of SMARCAL1-WT and SMARCAL1-ΔN. Top, quantifications (error bars indicate SEM of four replicates); bottom, representative experiment.

Supplementary Figure 3

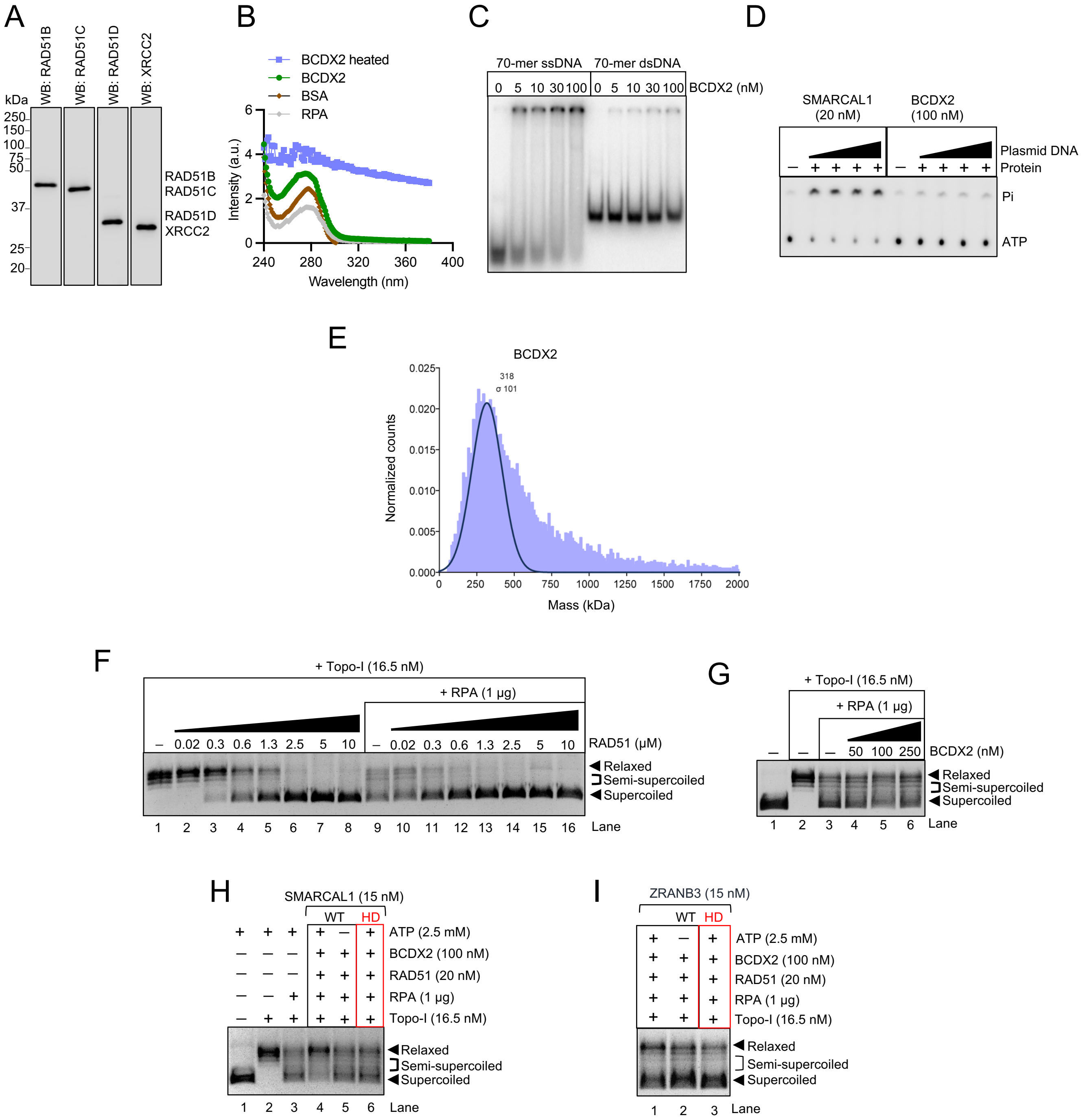

Supplementary Figure 3 (Related to Figure 3)

(A) Western blot analysis of the recombinant BCDX2 complex.

(B) Spectrophotometric analysis showing recombinant BCDX2 does not form aggregates. BSA and RPA were used as negative controls. However, when heated at 65 °C for 30 minutes, BCDX2 aggregated.

(C) Electrophoretic mobility shift assay showing BCDX2 has a much higher affinity towards 70-mer ssDNA (1 nM, in molecules) in contrast to 70-mer dsDNA (1 nM, in molecules).

(D) BCDX2 is a functional but weak ATPase. SMARCAL1 was used as a positive control. Plasmid DNA (2.961 kbp-long, 0.25, 0.5, 1 and 2 nM) was added as indicated in the figures.

(E) Mass photometry analysis of BCDX2 complex showing it is mostly a dimer of heterotetramer.

(F) and (G) Increasing concentration of RAD51 and BCDX2, respectively, does not promote annealing in the topoisomerase-coupled annealing assay.

(H) Topoisomerase-coupled annealing assay with wild type and helicase-dead (D549A, E550A, SMARCAL1-HD) SMARCAL1 variants. Shown is a representative experiment.

(I) Topoisomerase-coupled annealing assay with wild type and helicase-dead (D157A, E158A, ZRANB3-HD) ZRANB3 variants. Shown is a representative experiment.

Supplementary Figure 4

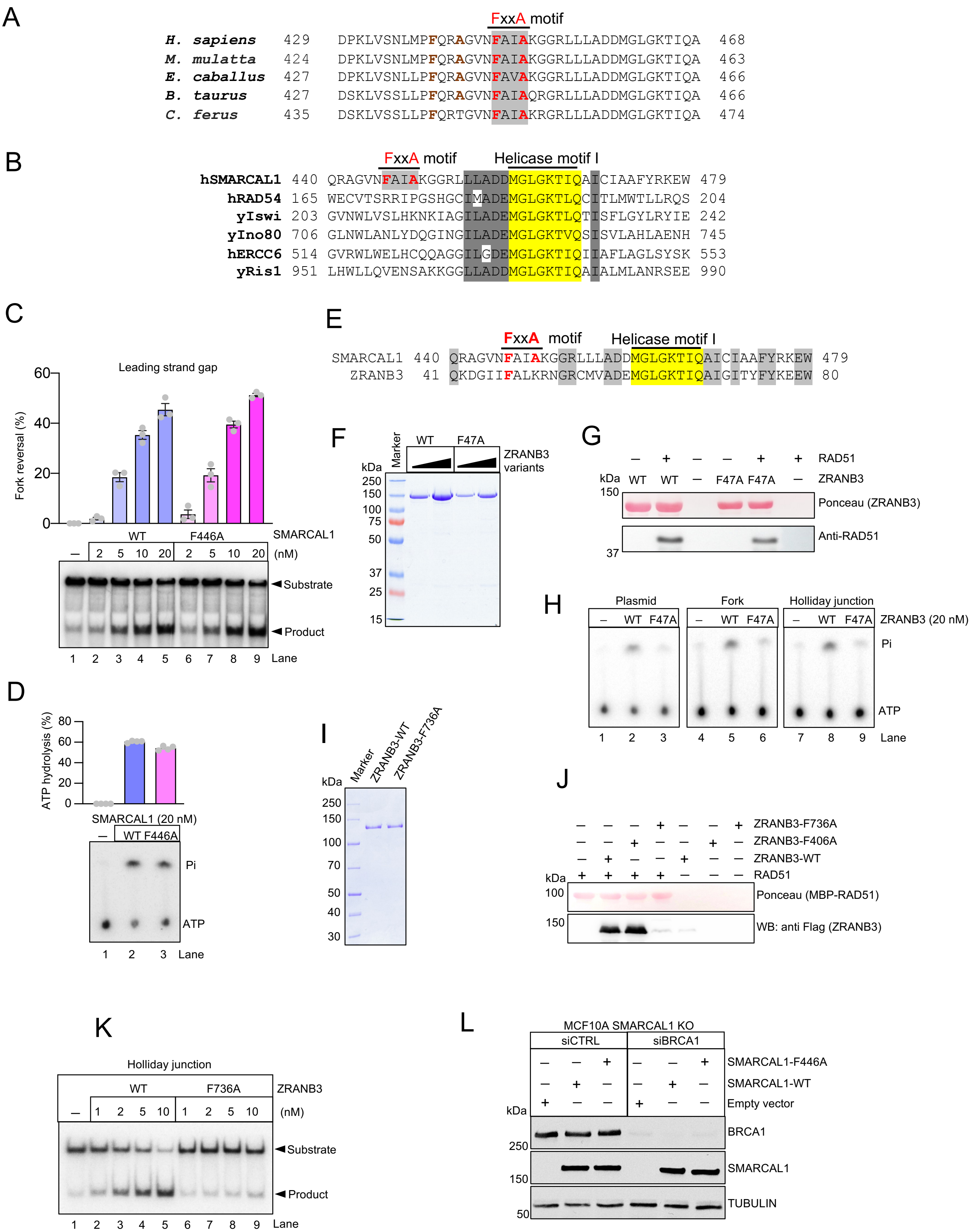

Supplementary Figure 4 (Related to Figure 5) . Legend on next page.

Supplementary Figure 4 (Related to Figure 5)

(A) Multiple sequence alignment showing conservation of the FxxA motif in SMARCAL1 in vertebrates, highlighted in grey with bold red letters. A less conserved second FxxA motif, with no effect on interaction with RAD51, is shown in bold brown letters.

(B) Multiple sequence alignment showing conserved helicase motif I (highlighted in yellow) in Snf2 family members in multiple yeast (y) and human (h) proteins. Additional conserved residues are highlighted in dark grey. The RAD51 interaction motif FxxA, indicated with bold red letters and highlighted in light grey in SMARCAL1, is located just upstream, but is not a part of the conserved ATPase domain.

(C) SMARCAL1-WT and SMARCAL1-F446A have comparable biochemical activities on a leading strand gap substrate. Note that the experiment was performed without RPA and in presence of 120 mM NaCl. Top, quantifications (error bars indicate SEM of three replicates); bottom, representative experiment.

(D) ATP hydrolysis by SMARCAL1-WT or SMARCAL1-F446A with a plasmid dsDNA as a substrate in the presence of 120 mM NaCl. Top, quantifications (error bars indicate SEM of four replicates); bottom, representative experiment.

(E) Sequence alignment showing conservation between human SMARCAL1 and ZRANB3, highlighted in grey. FxxA motif is highlighted in bold red letters in SMARCAL1. The corresponding phenylalanine (F) is also indicated in ZRANB3 in bold red letter.

(F) Recombinant ZRANB3-WT and ZRANB3-F47A were analyzed by polyacrylamide gel electrophoresis and stained with Coomassie Brilliant Blue.

(G) F47A substitution in ZRANB3 does not impair its interaction with RAD51. Soluble extract from *Sf*9 cells expressing FLAG-ZRANB3 variants (bait) was immobilized on M2 anti-FLAG affinity resin and tested for interaction with recombinant and purified RAD51 (prey). Samples were analyzed by polyacrylamide gel electrophoresis and either stained with Ponceau to show ZRANB3 or subjected to Western blot analysis with anti-RAD51 antibody.

(H) F47A substitution in ZRANB3 impairs ATP hydrolytic capacity with co-factors such as plasmid dsDNA (7 nM, in molecules), replication fork (5 nM) and Holliday junction (5 nM).

(I) Recombinant ZRANB3-WT and ZRANB3-F736A were analyzed by polyacrylamide gel electrophoresis and stained with Coomassie Brilliant Blue.

(J) Soluble extract from *E. coli* containing MBP-RAD51 (bait) was immobilized on amylose resin and incubated with purified recombinant ZRANB3-WT and ZRANB3-F736A (prey) and ZRANB3-F406A (prey). Samples were analyzed by polyacrylamide gel electrophoresis and either stained with Ponceau to show RAD51 or subjected to Western blot analysis with anti-FLAG antibody to detect ZRANB3.

(K) F736A substitution in ZRANB3 severely affects its branch migration activity. Shown is a representative experiment.

(L) Western blot analysis showing the efficiency of siRNA-mediated BRCA1 depletion and the comparable expression levels of SMARCAL1-WT or -F446A in SMARCAL1 KO cells, as indicated.

Supplementary Table 1
List of oligonucleotides used in this study

Primer Name	DNA sequence (5’-3’)
SMARCAL1 F439A F	ATTGACTCCAGCTCTCTGAGCGGGCATCAGATTAGACACG
SMARCAL1 F439A R	CGTGTCTAATCTGATGCCCGCTCAGAGAGCTGGAGTCAAT
SMARCAL1 F446A F	GCCCTTTCAGAGAGCTGGAGTCAATGCTGCCATAGCCAAA
SMARCAL1 F446A R	TTTGGCTATGGCAGCATTGACTCCAGCTCTCTGAAAGGGC
ZRANB3 F47A F	CTACTTCCATTCCAGAAAGATGGCATCATTGCTGCCCTCAAAAGAAAT
ZRANB3 F47A R	ATTTCTTTTGAGGGCAGCAATGATGCCATCTTTCTGGAATGGAAGTAGC
ZRANB3 F736A F	TCAGTATTCCTACTTGCACAGGCCATTAAGGTGTCATACACTGG
ZRANB3 F736A R	CCAGTGTATGACACCTTAATGGCCTGTGCAAGTAGGAATACTGA
SMARCAL1ΔN F	ATCGTACGACTAGTGGCGGAGAACAGCATCAGAGGACTAGC
SMARCAL1ΔN R	AGCAGGCTCGAGTTACAGGGGAGACGTAAAGCTGTC
XO2	TGGGTAAACCTGCAGGTGGGCAAAGATGTCCATCTGTTGTAATCGTCAAGCTTTATGCCGTT
XO1	GACGCTGCCGAATTCTACCAGTGCCTTGCTAGGACATcTTTGCCCACCTGCAGGTTCACCC
XO1C.MM2	GGGTGAACCTGCAGGTGGGCAAAAATGTCCTAGCAAGGCACTGGTAGAATTCGGCAGCGTC
XO2C.MM	GAACGGCATAAAGCTTGACGATTACAACAGATGGACATTTTTGCCCACCTGCAGGTTCACCC
#DC1	CGTGACTTGATGTTAACCCTAACCCTAAGATATCGCGTTATCAGAGTGTGAGGATACATGT<br>AGGCAATTGCCACGTGTCTATCAGCTGAAGTTGTTCGCGACGTGCGATCGTCGCTGCGACG
#DC2	CGTCGCAGCGACGATCGCACGTCGCGAACAACTTCAGCTGATAGACACGTGGCAATTGCCT<br>ACATGTATCCTCACACTCTGAATACGCGATATCTTAGGGTTAGGGTTAACATCAAGTCACG
#DC3	CGTCGCAGCGACGATCGCACGTCGCGAACAACTTCAGCTGATAGACACGTGGCAATTGCCT<br>ACATGTATCCTCACACTCTGA
#DC4	CGTCGCAGCGACGATCGCACGTCGCGAACAACTTCAGCTGATAGACACGTGG
#DC5	CCACGTGTCTATCAGCTGAAGTTGTTCGCGACGTGCGATCGTCGCTGCGACG
#DC6	TCAGAGTGTGAGGATACATGTAGGCAATTGCCACGTGTCTATCAGCTGAAGTTGTTCGCGAC<br>GTGCGATCGTCGCTGCGACG
X12-3	GACGTCATAGACGATTACATTGCTAGGACATGCTGTCTAGAGACTATCGC
X12-4C	GCGATAGTCTCTAGACAGCATGTCCTAGCAATGTAATCGTCTATGACGTC
PC-210	GTAAGTGCCGCGGTGCGGGTGCCAGGGCGTGCCCTTGGGCTCCCCGGGCGCGTACTCCAC<br>CTCATGCATC
PC-211	GATGCATGAGGTGGAGTACGCGCCCGGGGAGCCCAAGGGCACGCCCTGGCACCCGCACCG<br>CGGCACTTAC
