# Supplementary material for: Strand annealing and motor driven activities of SMARCAL1 and ZRANB3 are stimulated by RAD51 and the paralog complex
Source: Nucleic Acids Res. 2022 Jul 8;50(14):8008–22. doi: 10.1093/nar/gkac583 (PMC9371921; doi:10.1093/nar/gkac583)
Supplement: gkac583_Supplemental_Files [file gkac583_supplemental_files.zip › Uncropped Blots and Gels.pptx]

## Slide 1
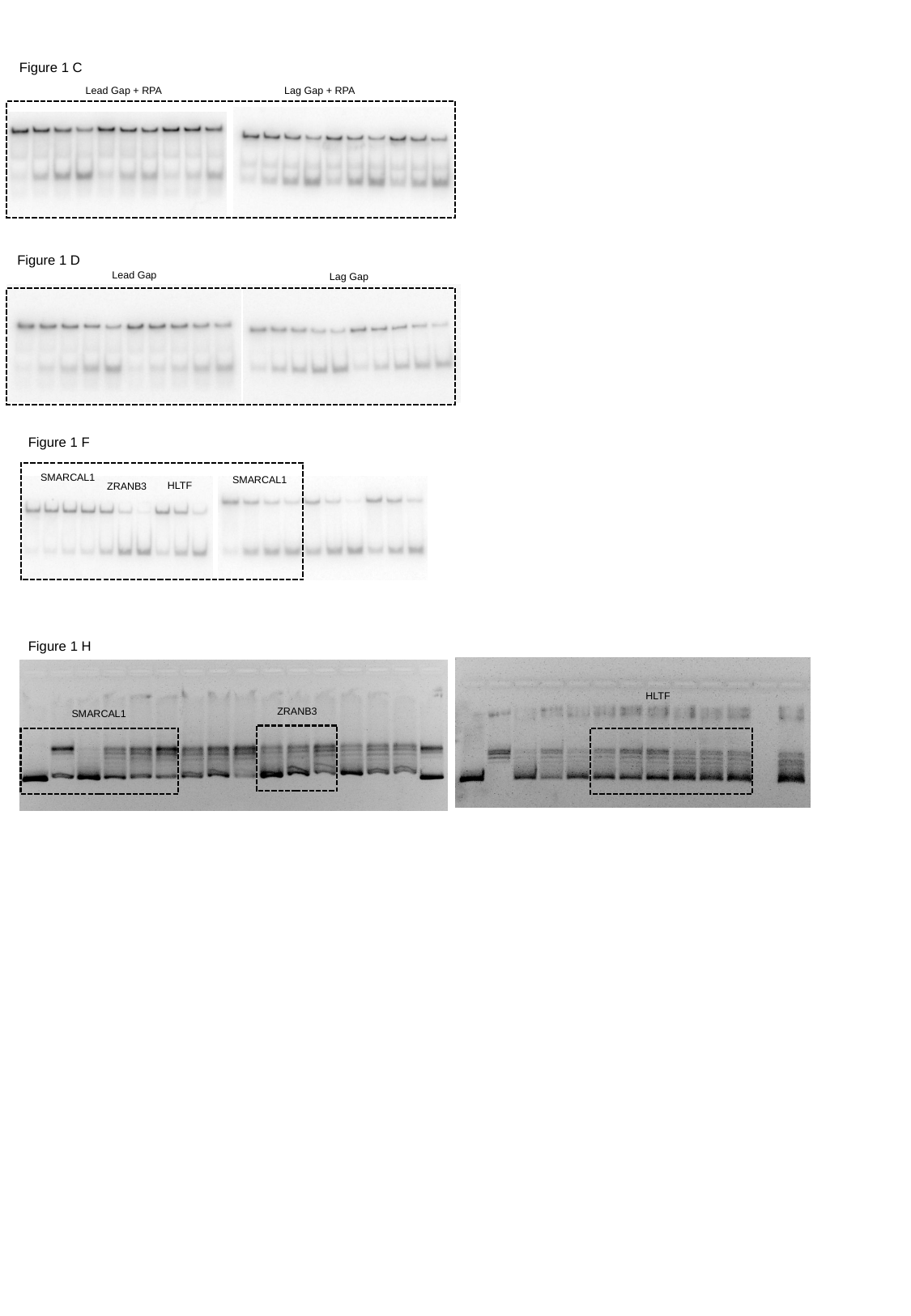

Figure 1 C
Lead Gap + RPA
Lag Gap + RPA
Figure 1 D
Lead Gap
Lag Gap
Figure 1 F
SMARCAL1
SMARCAL1
HLTF
ZRANB3
Figure 1 H
HLTF
ZRANB3
SMARCAL1

## Slide 2
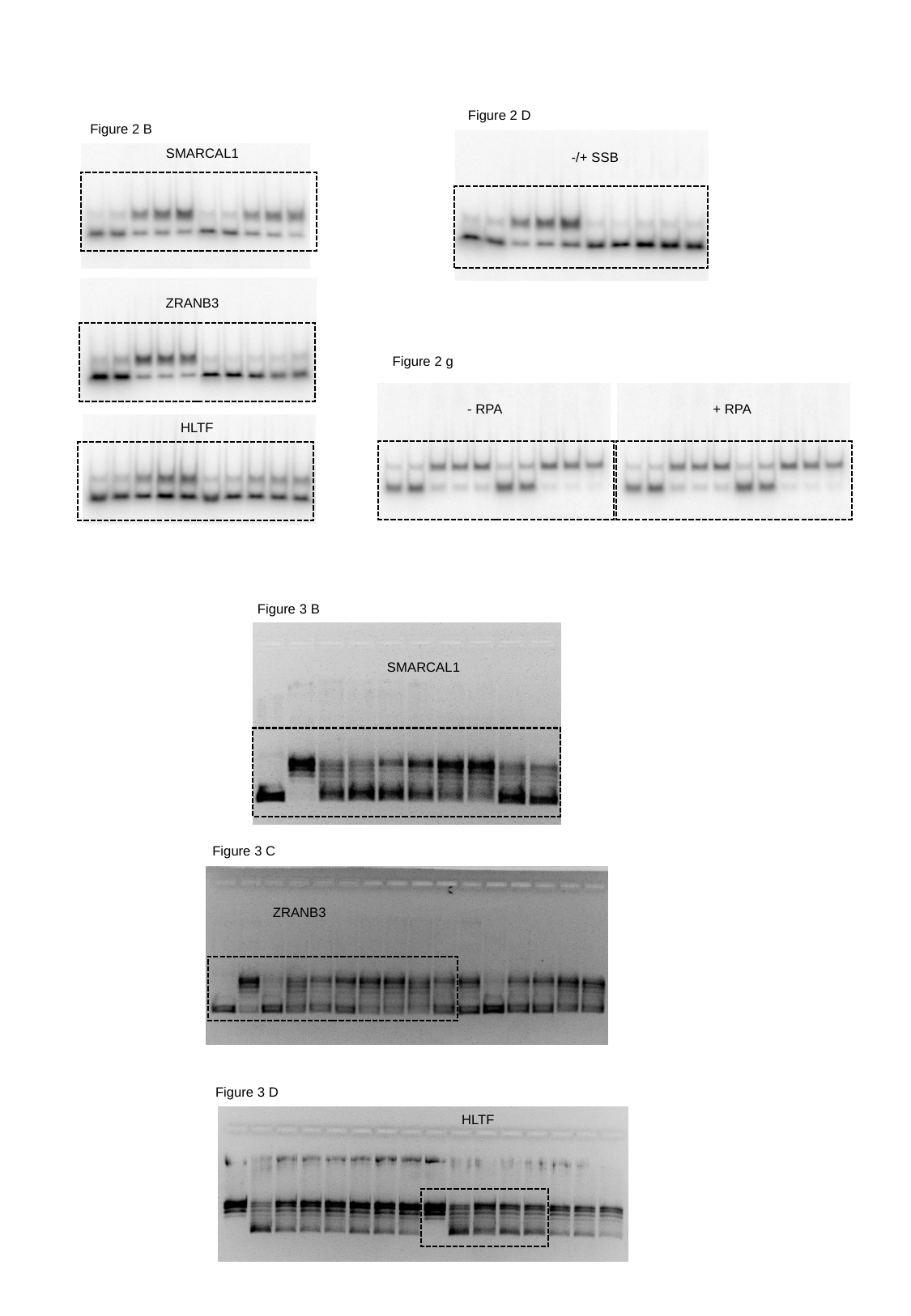

Figure 2 D
Figure 2 B
SMARCAL1
-/+ SSB
ZRANB3
Figure 2 g
- RPA
+ RPA
HLTF
Figure 3 B
SMARCAL1
Figure 3 C
ZRANB3
Figure 3 D
HLTF

## Slide 3
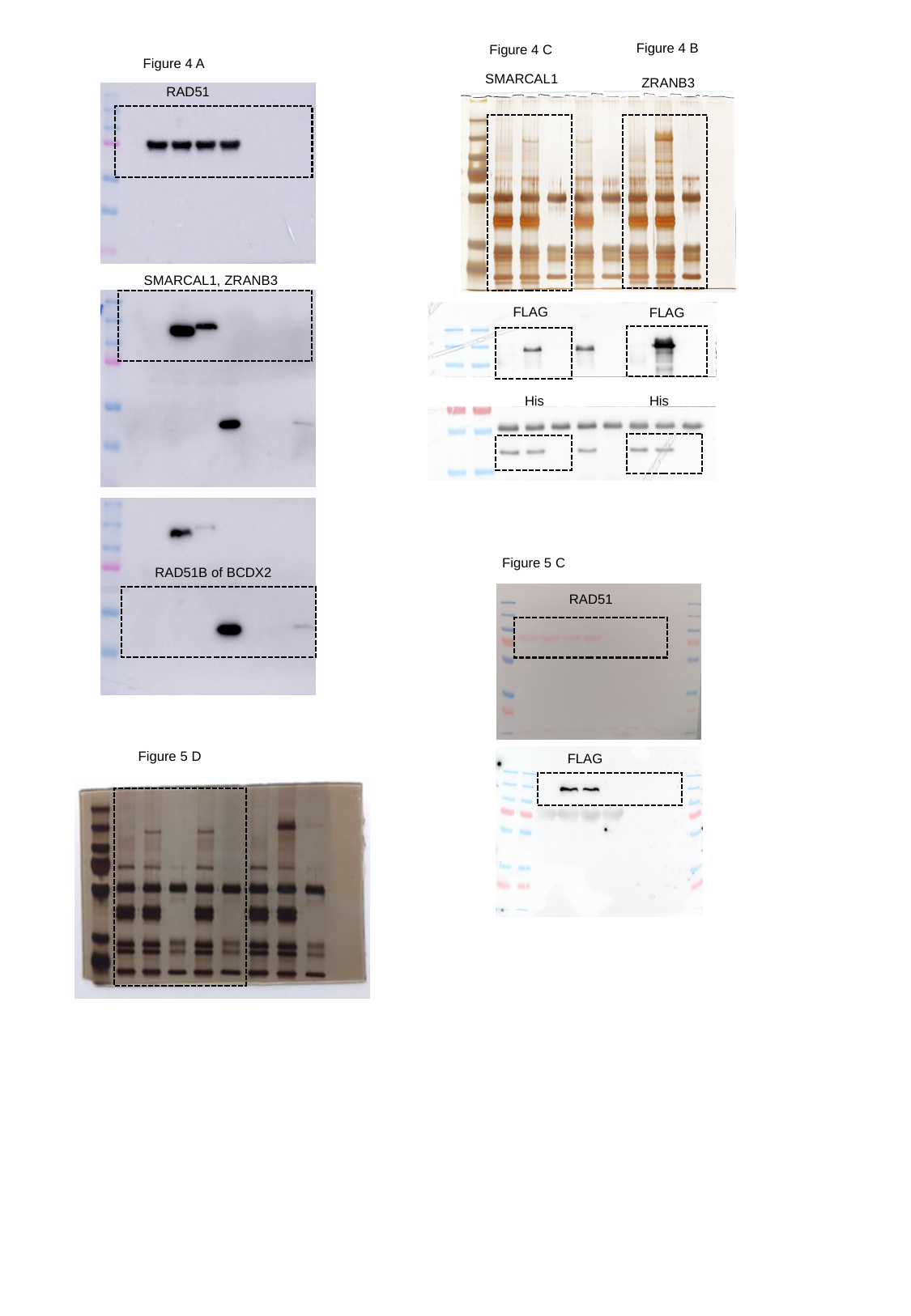

Figure 4 B
Figure 4 C
Figure 4 A
SMARCAL1
ZRANB3
RAD51
SMARCAL1, ZRANB3
FLAG
FLAG
His
His
Figure 5 C
RAD51B of BCDX2
RAD51
Figure 5 D
FLAG

## Slide 4
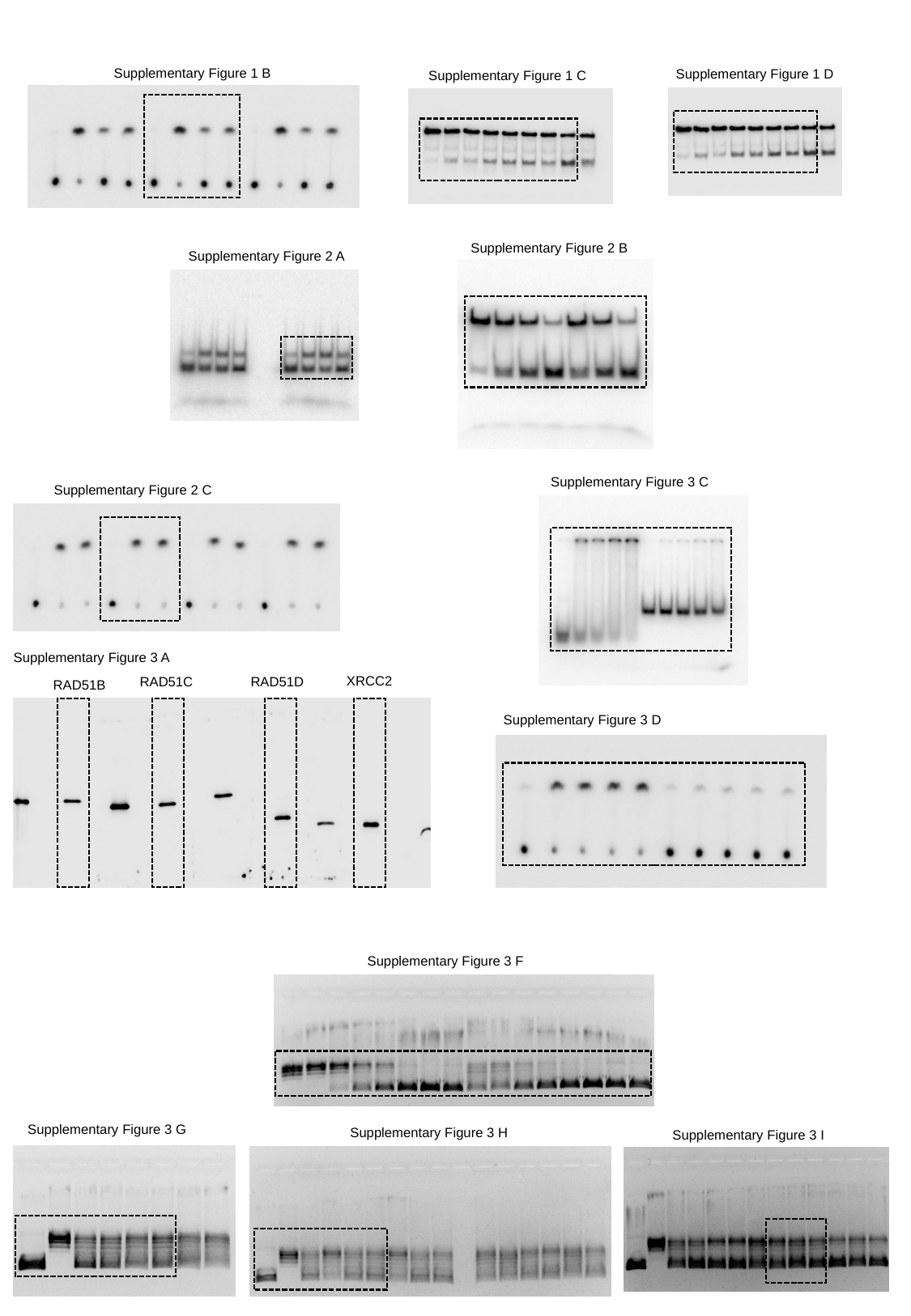

Supplementary Figure 1 B
Supplementary Figure 1 D
Supplementary Figure 1 C
Supplementary Figure 2 B
Supplementary Figure 2 A
Supplementary Figure 3 C
Supplementary Figure 2 C
Supplementary Figure 3 A
XRCC2
RAD51D
RAD51C
RAD51B
Supplementary Figure 3 D
Supplementary Figure 3 F
Supplementary Figure 3 G
Supplementary Figure 3 H
Supplementary Figure 3 I

## Slide 5
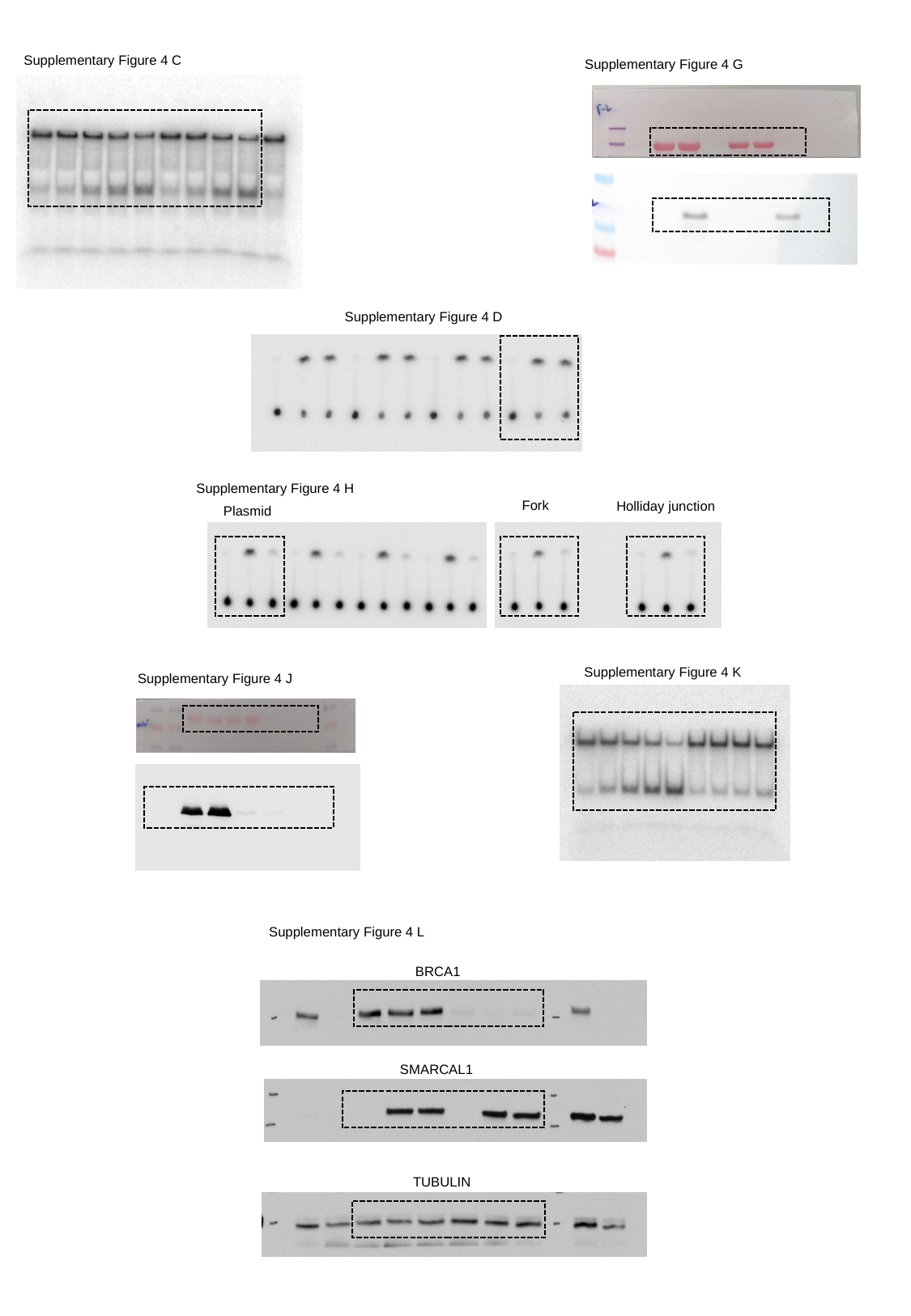

Supplementary Figure 4 C
Supplementary Figure 4 G
Supplementary Figure 4 D
Supplementary Figure 4 H
Fork
Holliday junction
Plasmid
Supplementary Figure 4 K
Supplementary Figure 4 J
Supplementary Figure 4 L
BRCA1
SMARCAL1
TUBULIN
